# Supplementary material for: The International Classification of Functioning, Disability and Health to map leprosy-related disability in rural and remote areas in Indonesia
Source: PLoS Negl Trop Dis. 2024 May 21;18(5):e0011539. doi: 10.1371/journal.pntd.0011539 (PMC11161106; doi:10.1371/journal.pntd.0011539)
Supplement: Attachment — Submitted filename: PNTD-D-23-00915_R1_reviewed.pdf [file pntd.0011539.s020.pdf]

PLOS Neglected Tropical Diseases

The International Classification of Functioning, Disability and Health to Map Leprosy-Related Disability in Rural and Remote Areas in Indonesia

--Manuscript Draft--

**Manuscript Number:**	PNTD-D-23-00915R1
**Full Title:**	The International Classification of Functioning, Disability and Health to Map Leprosy-Related Disability in Rural and Remote Areas in Indonesia
**Short Title:**	ICF to Map Leprosy-Related Disability in Indonesia
**Article Type:**	Research Article
**Keywords:**	Leprosy; ICF; disability; Lewoleba; Likupang
**Abstract:**	The International Classification of Function, Disability and Health (ICF - WHO, 2001) recognizes several dimensions of disability, such as body structure and function (and impairment thereof), activity (and activity restrictions) and participation (and participation restriction) and their interactions with contextual factor (personal and environmental). In this study, we map and analyse the relationship between the components of ICF in leprosy patients from two rural areas in Indonesia: Lewoleba (East Nusa Tenggara) and Likupang (North Minahasa). This study was a part of a community outreach program by the KATAMATAKU team from Universitas Indonesia. The body structure was graded using the WHO hand and feet disability grade and the number of enlarged nerves, whilst the body function was measured by the Jebsen Taylor Hand Function Test (JTT) and Timed-up and Go (TUG). Activity limitation and participation restriction were measured using the Screening Activity Limitation Safety Awareness (SALSA) Scale and Participation Scale (P-scale), respectively. There were 177 leprosy patients from the two regions and 150 patients with complete data were included in the analysis. We found 82% subjects with multibacillary leprosy, 10.67% subjects with grade 2 WHO hand disability, and 9.33% subjects with grade 2 WHO foot disability. Assessment using SALSA Scale showed 29.33% of subjects with limitation activity and 11.33% with participation restriction. Age was shown to have positive correlations with SALSA, JTT, and TUG. Inter-dimensional analysis showed that the SALSA scale had significant positive correlations with the number of nerve enlargement, P-scale, JTT, and TUG. SALSA scores of grade 2 WHO hand and foot disability were also significantly higher than grade 1 and 0. Participation scale also had a positive correlation with JTT but not TUG. Hand disability seemed to affect societal participation whilst foot did not. We used the ICF to describe and analyse dimensions of leprosy-related disability in Indonesia.
**Additional Information:**	
**Question**	**Response**
**Government Employee**<br><br>Are you or any of the contributing authors an employee of the United States government?<br><br>Manuscripts authored by one or more US Government employees are not copyrighted, but are licensed under a CC0 Public Domain Dedication, which allows unlimited distribution and reuse of the article for any lawful purpose. This is a legal requirement for US Government employees.	No - No authors are employees of the U.S. government.

Powered by Editorial Manager® and ProduXion Manager® from Aries Systems Corporation

This will be typeset if the manuscript is accepted for publication.	
**Financial Disclosure**<br><br>Enter a financial disclosure statement that describes the sources of funding for the work included in this submission. Review the submission guidelines for detailed requirements. View published research articles from *PLOS Neglected Tropical Diseases* for specific examples.<br><br>This statement is required for submission and **will appear in the published article** if the submission is accepted. Please make sure it is accurate.<br><br>**Funded studies**<br>Enter a statement with the following details:<br>• Initials of the authors who received each award<br>• Grant numbers awarded to each author<br>• The full name of each funder<br>• URL of each funder website<br>• Did the sponsors or funders play any role in the study design, data collection and analysis, decision to publish, or preparation of the manuscript?<br><br>Did you receive funding for this work?	Yes
Please add funding details.<br>as follow-up to "**Financial Disclosure**<br><br>Enter a financial disclosure statement that describes the sources of funding for the work included in this submission. Review the submission guidelines for detailed requirements. View published research articles from *PLOS Neglected Tropical Diseases* for specific examples.<br><br>This statement is required for submission and **will appear in the published article** if the submission is accepted. Please make sure it is accurate.	This study was funded by grant from Directorate of Research and Development, Universitas Indonesia under International-Indexed Publication Grant (PUTI) 2022 (Grant No: NKB-1402/UN2.RST/HKP.05.00/2022), with the recipient of our author LKW. Funder data could be accessed from https://ppm.ui.ac.id. The funders had no role in study design, data collection and analysis, decision to publish, or preparation of the manuscript.

**Funded studies**<br>Enter a statement with the following details:<br>• Initials of the authors who received each award<br>• Grant numbers awarded to each author<br>• The full name of each funder<br>• URL of each funder website<br>• Did the sponsors or funders play any role in the study design, data collection and analysis, decision to publish, or preparation of the manuscript?<br><br>Did you receive funding for this work?"	
Please select the country of your main research funder (please select carefully as in some cases this is used in fee calculation).<br>as follow-up to "**Financial Disclosure**<br><br>Enter a financial disclosure statement that describes the sources of funding for the work included in this submission. Review the submission guidelines for detailed requirements. View published research articles from *PLOS Neglected Tropical Diseases* for specific examples.<br><br>This statement is required for submission and **will appear in the published article** if the submission is accepted. Please make sure it is accurate.<br><br>**Funded studies**<br>Enter a statement with the following details:<br>• Initials of the authors who received each award<br>• Grant numbers awarded to each author<br>• The full name of each funder<br>• URL of each funder website<br>• Did the sponsors or funders play any role in the study design, data collection and analysis, decision to publish, or preparation of the manuscript?<br><br>Did you receive funding for this work?"	INDONESIA - ID

Powered by Editorial Manager® and ProduXion Manager® from Aries Systems Corporation

**Competing Interests**<br><br>On behalf of all authors, disclose any competing interests that could be perceived to bias this work.<br><br>This statement will be typeset if the manuscript is accepted for publication.<br><br>Review the instructions link below and PLOS NTDs' competing interests policy to determine what information must be disclosed at submission.	This study has no competing interest
**Data Availability**<br><br>Provide a **Data Availability Statement** in the box below. This statement should detail where the data used in this submission can be accessed. This statement will be typeset if the manuscript is accepted for publication.<br><br>Before publication, authors are required to make all data underlying their findings fully available, without restriction. Review our PLOS Data Policy page for detailed information on this policy. Instructions for writing your Data Availability statement can be accessed via the Instructions link below.	All relevant data are within the manuscript and its supporting information files.

Powered by Editorial Manager® and ProduXion Manager® from Aries Systems Corporation

Manuscript Revised
Click here to access/download;Manuscript;Manuscript (unmarked)(1).docx

The International Classification of Functioning, Disability and Health to map leprosy-related disability in rural and remote areas in Indonesia

Luh Karunia Wahyuni[*1¶], Nelfidayani[1¶], Melinda Harini[1¶], Fitri Anestherita[1¶], Rizky Kusuma Wardhani[1¶], Sri Linuwih Menaldi[2], Yunia Irawati[3], Tri Rahayu[3], Gitalisa Andayani[3], Hisar Daniel[3], Intan Savitri[1], Petrus Kanisius Yogi Hariyanto[1], Isabela Andhika Paramita[1,4]

Affilitation(s)

[1] Department of Physical Medicine and Rehabilitation, Faculty of Medicine Universitas Indonesia, dr. Cipto Mangunkusumo Hospital, Jakarta, Indonesia

[2] Department of Dermatology and Venereology, Faculty of Medicine Universitas Indonesia, dr. Cipto Mangunkusumo Hospital, Jakarta, Indonesia

[3] Department of Ophthalmology, Faculty of Medicine Universitas Indonesia, dr. Cipto Mangunkusumo Hospital, Jakarta, Indonesia

[4] Genomik Solidaritas Indonesia (GSI) Laboratory, Jakarta, Indonesia

* Corresponding author

E-mail: luhkwahyuni@gmail.com

[¶] These authors contributed equally to this work.

Abstract

The International Classification of Function, Disability and Health (ICF - WHO, 2001) recognizes several dimensions of disability, such as body structure and function (and impairment thereof), activity (and activity restrictions) and participation (and participation restriction) and their interactions with contextual factor (personal and environmental). In this study, we map and analyse the relationship between the components of ICF in leprosy patients from two rural areas in Indonesia: Lewoleba (East Nusa Tenggara) and Likupang (North Minahasa). This study was a part of a community outreach program by the KATAMATAKU team from Universitas Indonesia. The body structure was graded using the WHO hand and feet disability grade and the number of enlarged nerves, whilst the body function was measured by the Jebsen Taylor Hand Function Test (JTT) and Timed-up and Go (TUG). Activity limitation and participation restriction were measured using the Screening Activity Limitation Safety Awareness (SALSA) Scale and Participation Scale (P-scale), respectively. There were 177 leprosy patients from the two regions and 150 patients with complete data were included in the analysis. We found 82% subjects with multibacillary leprosy, 10.67% subjects with grade 2 WHO hand disability, and 9.33% subjects with grade 2 WHO foot disability. Assessment using SALSA Scale showed 29.33% of subjects with limitation activity and 11.33% with participation restriction. Age was shown to have positive correlations with SALSA, JTT, and TUG. Inter-dimensional analysis showed that the SALSA scale had significant positive correlations with the number of nerve enlargement, P-scale, JTT, and TUG. SALSA scores of grade 2 WHO hand and foot disability were also significantly higher than grade 1 and 0. Participation scale also had a positive correlation with JTT but not TUG. Hand disability seemed to affect societal participation whilst foot did not. We used the ICF to describe and analyse dimensions of leprosy-related disability in Indonesia.

Keywords: leprosy, ICF, disability, Lewoleba, Likupang

Author Summary

Disability is the long-term outcome of untreated leprosy or Hansen's disease, which is caused by peripheral nerve invasion of the *Mycobacterium leprae*. It is a serious and life-limiting complication to leprosy patients. Currently, there are seven regions in Indonesia which have not yet achieved the state of disease elimination. In addition, Indonesia has not succeeded in achieving the sub-target of rate of new cases with grade 2 disability in 2023. The concept of disability and health including the body structure, body function, personal and environmental domains, activity limitation, and participation restriction, is provided by the ICF. Through the framework, we could assess the aspects of disability and its relationship to one another in leprosy patients. Furthermore, the early detection, screening, and management programs for leprosy have not included aspects of disability and rehabilitative measures for restoration. Thus, the purpose of this study is to find the impact of leprosy-related disability in the patients' body function, activity, and participation. We report 150 leprosy patients from two rural areas of Indonesia and found significant relationships between specific indicators, which would be useful for rehabilitative management programs in the future.

Introduction

Infiltration of *Mycobacterium leprae* into Schwann cells in leprosy can cause peripheral nerve inflammation and subsequent progressive loss of nerve fibre function or neuropathy [1]. This condition mainly affects the hands (44.45%), feet (39,76%), and face (15,74%) [2]. The subsequent loss of sensory; motor; and autonomic nerve function would appear as loss of thermal, nociceptive, and pressure senses; muscle paresis; and dryness of the skin, respectively. Occasionally, delayed emergence of skin lesions and sensory impairment may also occur, termed as silent neuropathy [1]. When left untreated, these conditions can develop into visible

physical deformities (lagophthalmos, severe visual impairments, ulcers, clawing and shortening of digits in the extremities), which the World Health Organization (WHO) classify as grade 2 disabilities [3]. Unfortunately, the majority of newly diagnosed leprosy patients have already had deformities [2].

Disabilities in leprosy patients cause physical disability, psychological disturbances, and extensive loss of manpower and economic loss to the society [3]. Assessment of disability in leprosy patients is a very important factor in the evaluation of the effectiveness of a leprosy elimination program [3]. Disability can be perceived as any impairment of a person's health and mind condition that renders the person to have functioning difficulty at the body, person, or societal levels, in any life domain [4,5]. The International Classification of Functioning, Disability and Health (ICF) recognizes dimensions of disability, which include body structure and function (along with each impairments), activity and limitations, as well as participation and restrictions. Environmental aspects of the individual are also emphasized as a major predictor of disability outcomes thereby shifting the paradigm of disability as a mere 'medical' or 'biological' dysfunction [4].

Along with India and Brazil, Indonesia is one of the major leprosy contributors in the world. In the 2021 global leprosy update by WHO, the majority of new case detection came from South-East Asia and more than 10,000 cases were found in Indonesia [6]. According to the Indonesian Ministry of Health report, leprosy cases are still increasing annually, from 5 cases per 100.000 population in 2021 to 5.5 cases per 100.000 population in 2022 [7]. Recent reports have shown that the number of leprosy patients with grade 2 WHO disability has widely ranged from 6.31% to 68.2% [2,8,9]. As of 2021, there were 12,230 registered leprosy cases in Indonesia, with a new case detection rate of 4.03 per 100,000 population and 10,976 new cases reported. The proportion of new leprosy cases with grade 2 deformities was determined to be 2.46 per 1,000,000 population, which still surpasses the WHO's target of 0.92 per 1,000,000

population by 2023 [10,11]. However, studies related to the burden of leprosy are fewer than those related to endemicity [12]. Not to mention that research pertaining to the specific challenges encountered by leprosy-related disabilities patients in Indonesia are very scarce. These data are urgently needed for initiating sustainable rehabilitative measures for disability prevention and for improving the patients' well-being.

The objectives of this study are to report leprosy cases from rural and remote areas in Indonesia, map the functional disabilities of individuals diagnosed with leprosy in Likupang and Lewoleba, Indonesia, using ICF, and to find correlation between each domain of ICF in Indonesian population. We believe that there was intercorrelation among aspects of ICF in the Indonesian leprosy population.

Materials and methods

Ethical aspects

The study protocol was approved by the Ethics Committee of Faculty of Medicine, Universitas Indonesia under ethical clearance ND-454/UN2.F1/ETIK/PPM.00.02/2022 and was designed in consideration of the principles proposed by the Helsinki Declaration. Written formal consent was obtained directly from participants.

Study design, period, and location

This cross-sectional data was obtained from a community outreach program by KATAMATAKU that was carried out in Lewoleba, Lembata, East Nusa Tenggara (July 2022) and Likupang, North Minahasa, North Sulawesi (August 2022). The KATAMATAKU team is a multidisciplinary (expert) team made up of dermatologists, ophthalmologists and physical medicine and rehabilitation (PMR) specialists from Faculty of Medicine Universitas Indonesia in Jakarta, Indonesia. The word KATAMATAKU is an Indonesian acronym for '*Identifikasi*

tanda-tanda mata, ekstremitas dan kulit pada kusta' or identification of ocular, extremities, and dermatological signs in leprosy. The collaborative team often visits remote sites with higher leprosy-population density to do surveillance and charitable community engagements with leprosy patients and survivors. The outreach program consisted of physical examination, consultation, and treatment for leprosy patients.

The study participants from Likupang, North Minahasa and Lewoleba, Lembata were those who attended the events held in July 2022 and August 2022, respectively. The individuals participating in our investigation received their diagnoses in accordance with the diagnostic criteria for leprosy established by the World Health Organization (WHO). Present-day leprosy diagnosis relies on the identification of at least one of the three primary diagnostic indicators: (i) pronounced sensory loss within a pale (hypopigmented) or reddish skin region; (ii) discernible thickening or enlargement of a peripheral nerve accompanied by sensory loss and/or muscle weakness innervated by that nerve; or (iii) the detection of acid-fast bacilli in a slit-skin smear. [13].

Additionally, we also contacted the local public health officers a month prior to the event and the affiliated regional hospitals and primary health care facilities notified the residing population nearby and registered leprosy patients. Patients were referred by St. Damian Hospital (Lewoleba) and the public health office (Likupang) to attend our community outreach events in the respective months (July and August 2022).

Lewoleba, which is the capital of Lembata island, East Nusa Tenggara, is in the Nubatukan district. Lewoleba has an area of 165.64 KM^2 whilst Lembata spans over 1.266,4 KM^2. Lembata island is divided into nine districts and has overall a tropical climate with a long drought. Lembata has relatively high humidity at around 80% [14]. Nubatukan district has the highest population density at 50.984 or 37% of total population of the island. Around 11.4%

of total population are aged 0-4 years. There are three hospitals and one public health center in Nubatukan [15]. The population in Lembata mostly work in agriculture, forestry, hunting and fisheries sectors [15]. East Nusa Tenggara is considered as one of the provinces in Indonesia with high leprosy incident, with around 7 per 100.000 people and high chance of getting disability due to leprosy about 27.78% [15,16].

Likupang region is further divided into three subdistricts: East Likupang, West Likupang and South Likupang. All of them are coastal sub districts located at 1.6720° N, 125.0553° E, 0 meters above the sea level, with a total area of 298.27 square kilometer ($KM^{2)}$ [17-19]. Since Likupang is located near the seashore, it has high humidity, reaching 80-90% on average [20]. In 2022, the region had 44.660 inhabitants, with average population growth rate of 1.39% between the years 2020 and 2022. South Likupang is the subdistrict with highest population density, while East Likupang is the lowest [21]. According to data from Ministry of Health in 2021, North Sulawesi is one of Provinces in Indonesia with high leprosy cases, with detection rate of 11 per 100,000 inhabitants in 2018 [22,23]. As of 2022, North Sulawesi is one of the 7 provinces where leprosy has not yet been eliminated. Likupang itself has 4 public health centers, 3 with inpatient care, as well as 3 clinics spread over three subdistricts [17-19]. The map of Lewoleba, Lembata, and Likupang, North Minahasa, can be found in **Figure 1**.

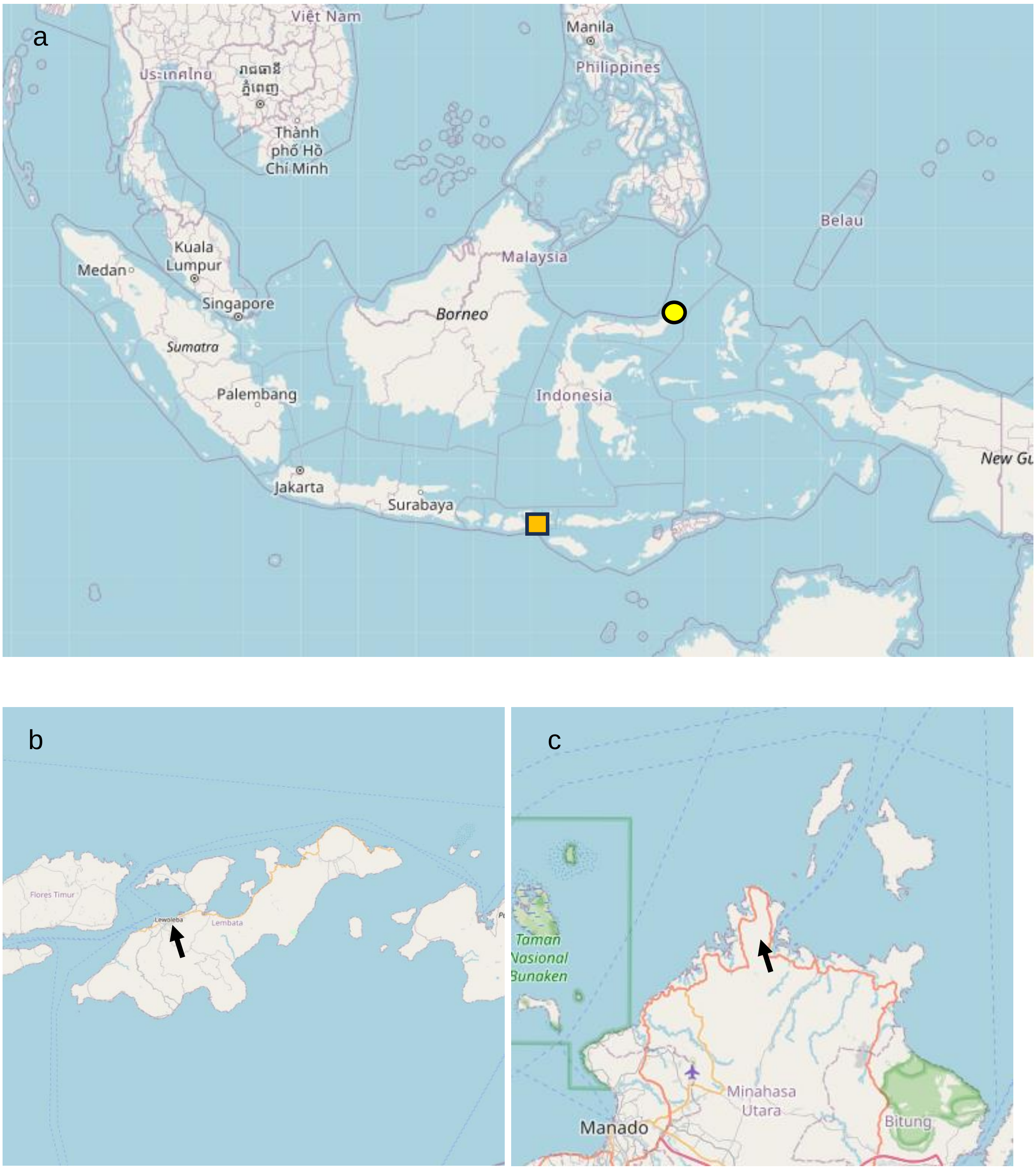

Figure 1. Map of Lewoleba (Lembata, East Nusa Tenggara) and Likupang (North Minahasa, North Sulawesi), Indonesia. (A) Map of Indonesia, scaled 1: 26500000 (753x477) (available from: https://www.openstreetmap.org/#map=5/0.637/117.466&layers=G, Geo URI: geo:0.681,117.510?z=5), yellow circle indicates Likupang, North Minahasa and orange rectangle indicates Lewoleba, Lembata. (B) Map of Lembata Island (East Nusa Tenggara), scaled 1: 547767 (789 x 721) (available from: https://www.openstreetmap.org/#map=10/-8.4078/123.6017&layers=G, Geo

URI: geo:-8.3732,123.4369?z=10), black arrow indicates Lewoleba. (C) Map of North Minahasa (North Sulawesi), scaled 1: 547767 (395 x 360) (available from: https://www.openstreetmap.org/?mlat=1.6961&mlon=125.0011#map=11/1.6961/125.0011&layers=G, Geo URI: geo:1.6960,125.0011?z=13), black arrow indicates Likupang. The map contains information from OpenStreetMap (OSM) and OpenStreetMap Foundation (accessed online on 16th of October 2023), which is made available under the Open Database License (Copyright and license: https://www.openstreetmap.org/copyright). Documentation of OSM is licensed under the Creative Commons Attribution-ShareAlike 2.0 license (CC BY-SA 2.0). Manual markings were done to locate Lewoleba and Likupang (Figure 1A).

Study protocol and population

In both Likupang and Lewoleba, information was gathered through the examination of all leprosy patients. All subjects who agreed to participate in the study filled the informed consent form. For patients below 18 years old, the informed consent forms were filled by parents or legal guardian. The participants were then provided with a set of documents consisting of SALSA and P-scale questionnaires. Subsequently, patients were directed to independently complete these forms, with the option of seeking assistance from staff members for clarifications if needed. Additionally, they were encouraged to use only their initials, rather than their full names, to ensure the confidentiality of their identities. These completed forms were then collected and securely stored at the University of Indonesia. Our minimum requirement of samples were calculated using the expected correlation coefficient [24] as follows;

$$N = \frac{(Z_{\frac{\alpha}{2}} + Z_{1-\beta})^2}{1/4[\log\left(\frac{1+r}{1-r}\right)]} + 4$$

with $Z_{\frac{\alpha}{2}}$=1.96, $Z_{1-\beta}$=0,84, and r=0.8. We discovered that the required N=33.925 or 34 patients.

The inclusion and exclusion criteria for the data used in our research are the following:

Inclusion criteria:

1. The patients are diagnosed with leprosy according to WHO criteria and referred from local hospitals and primary health care.
2. The study population all consists of local residents.
3. The patient provided written consent to take part in this research.

Exclusion criteria:

1. Patients with neuropathic disabilities unrelated to leprosy.
2. The presence of pre-existing disabilities.
3. The presence of mental disabilities.
4. Patients who refused to undergo examinations for body function (Jebsen-Taylor Hand Function Test (JTT) and timed up and go (TUG) test), activity limitation (SALSA), and participation restriction (P-scale).

Age, gender, and background were among the basic demographic data collected. Each participant filled out the SALSA questionnaire as well as the participation scale (P-scale) and went through the entire assessment process, including the timed up and go (TUG) test and the Jebsen-Taylor Hand Function Test (JTT). The SALSA questionnaire measures the activity limitation of the patients that consists of 20 questions related to ability to do daily activities and the three domains used in this study are mobility, self-care, and work [25]. The participation scale consists of 18 questions related to social participations of the patients (e.g. working, socializing with other people) [26]. These questionnaires were available in Indonesian

language [25, 26] and have been performed in Indonesian population prior of the study [8, 27, 28].

The study variables or assessment tools along with each description are listed in **Table 1**. The outcomes were analysed in accordance with the International Classification of Functioning, Disability and Health (ICF) domains as listed in **Figure 2**.

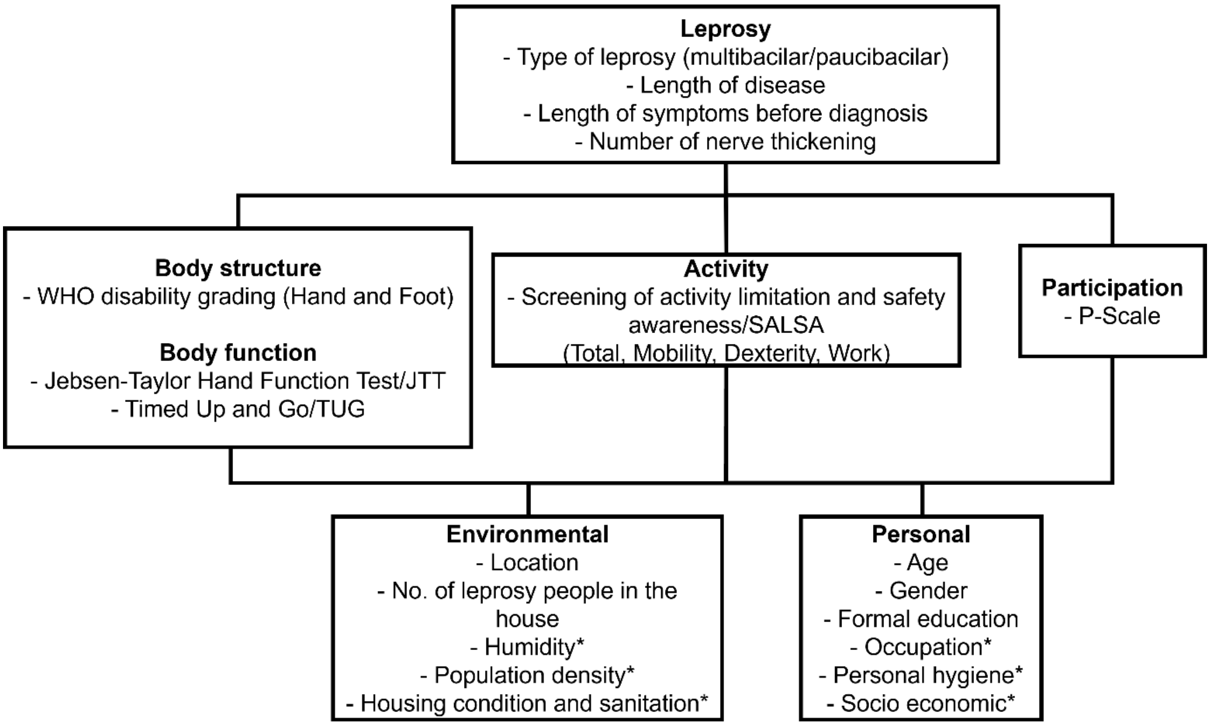

Figure 2. Application of the International Classification of Functioning, Disability and Health (ICF) domains in the study.[29] The domains of ICF have been adjusted to the availability of data within both populations in Likupang and Lembata. Asterisks indicate that the data was not available in our population. SALSA, screening of activity limitation and safety awareness; JTT, Jebsen-Taylor hand function test; TUG, timed up and go test.

Table 1. Assessment Tools according to the domains of International Classification of Functioning, Disability, and Health (ICF).

Item	Content	Evaluation	Note
Personal factors			
- Age		Identity card	<18 years old, 18-59 years old, ≥60 years old
- Gender		Identity card	Female, male
- Formal education level		Question to the patient	- Uneducated<br>- Primary Education<br>- Middle School<br>- Secondary Education

			- Higher Education
Health condition			
- Diagnosis of Leprosy		Anamnesis related to history of the disease, type of leprosy, and medication	Paucibacillary, multibacillary
- Number of person(s) living together with the same diagnosis		Anamnesis of any contacts within family members and whether they live together or separately with the patient	Number of family member with similar symptoms or diagnosis
Body structure			
- Physical Examinations	Abnormalities and deformities in the skin and extremities	- · Inspection of lesions and deformities<br>- · Motor and sensory nerve function	Number of nerve thickening.
- The WHO classification of physical disability in leprosy [30].		The presence of nerve impairments or visible deformity	Grade 0: no disability (no loss of sensitivity and no visible deformity or damage to the eyes, hands or feet)<br><br>Grade 1: only disability (a loss of sensitivity without visible deformity or damage to the eyes, hands or feet)<br><br>Grade 2: the presence of visible deformity or damage to the eyes (lagophthalmos, iridocyclitis, corneal opacities, severe visual impairment), hands (claw hands, ulcers, absorption of the digits, thumb-web contracture and swollen hand), feet (plantar ulcers, footdrop, inversion of the foot, clawing of the toes, absorption of the toes, collapsed foot and callosities)

Body function			
- The Jebsen-Taylor Hand Function Test (JTT) [31].	A standardized and objective measure of fine and gross motor hand function using simulated activities of daily living (ADL)	A series of seven subtests representing fine motor, non-weighted and weighted hand function in ADL, which includes: writing, simulated page-turning, lifting small objects, simulated feeding, stacking, and lifting large, lightweight, and heavy objects.	The subtests are scored by recording the number of seconds required to complete each test<br><br>Total score is the sum of time taken for each sub-test, which are rounded to the nearest second. Shorter times indicate better performance.
- Timed Up and Go Test (TUG) [32].	To determine the patient's fall risk and measure the progress of balance, sit to stand and walking	Assessed domains<br>● Balance<br>● Gait<br>● Walking speed	Patients performed the test one time, and if a clear error was made, they were asked to repeat the TUG.
Activity			
- The Screening Activity Limitation Safety Awareness (SALSA) Scale [25,33].	Includes 20 daily activities questions related to the three areas of mobility, self-care, and work.	The ability of patients to do basic daily activities such as walking, self-care, carrying heavy objects and manipulating small objects.	SALSA's Indonesian edition was used for this investigation. The total score ranges from 0-80, with the result classified as:<br>● no substantial limitation (0–24)<br>● mild limitation (25–39)<br>● moderate limitation (40–49)<br>● severe limitation (50–59)<br>● extreme limitation (60–80)
Participation			
- Participation Scale (P-Scale) [34].	Consists of 18 items to measure (social) participation for use in rehabilitation, stigma reduction and social integration programmes.	Assesses domains of participation including:<br>● Communication<br>● Mobility<br>● Self-Care<br>● Domestic Life<br>● Interpersonal Interactions<br>● Major life areas (work, education, etc)	The P-scale total score varies between 0-90, with the result classified as:<br>● No significant restriction (0-12)<br>● Mild restriction (13-22)<br>● Moderate restriction (23-32)<br>● Severe restriction (33-52)

		• Community, Social and Civic	• Extreme restriction (53-90)

Analysis of results and statistics

Microsoft Excel was used to store, organize, clean raw data and perform F tests for equal variance. Statistical analysis was performed using IBM Statistical Package for Social Sciences (SPSS) Statistics Software version 25.0 (IBM Corp., Armonk, NY) and figures were generated using GraphPad Prism version 10.0.2 (GraphPad Software, San Diego, CA). Normality and F tests were performed to all sets of data. For the comparison test between two groups, non-normal groups were analysed non-parametrically using Mann-Whitney U test and presented with median ± range. Normally distributed groups with equal and non-equal variances were compared using independent *t* test (presented with mean ± standard deviation (SD)) and Welch's *t* test (presented with mean), respectively. For the comparison between more than two groups, groups with non-normal distribution were analysed using Kruskal-Wallis test. Bivariate analyses using Pearson's and Spearman's correlation tests were performed in normally and non-normally distributed variables, respectively. Correlation coefficients below 0.3 were considered independent [35]. Heatmap was created using RStudio using the 'lattice' package along with 'cor' and 'levelplot' function. Categorical variables were analysed using Fisher's Exact and Chi-Square tests, and binary logistic regression analysis was further performed to statistically significant results with subsequent adjustment of confounders (presented with Odds Ratio (OR) and p-values). Confounders considered in this study include age (juvenile or ≤ 18 years, adult (18-59 years), and old (≥ 60 years)), gender (male and female), educational background (uneducated, primary education, middle school, secondary education, and higher education), location (Likupang and Lewoleba), occurrence of nerve thickening (none and present), length of disease (< 24 (median) months and ≥ 24 months), length of complaint before diagnosis (< 6 (median) months and ≥ 6 months), type of leprosy

(paucibacillary and multibacillary), hand and foot WHO disability (grade 0, grade 1, and grade 2), SALSA categories (no limitation, mild limitation, moderate limitation, severe limitation, and extreme limitation), P-scale categories (no restriction, mild restriction, moderate restriction, severe restriction, and extreme restriction), JTT in dominant hand (<20.94 (median) s and ≥ 20.94 s) and nondominant hand (<24.88 (median) s and ≥ 24.88 s), and TUG (< 13.5 s and ≥ 13.5 s). Probability of <0.05 is considered statistically significant.

Results

The total number of patients examined during the events were 177 patients with leprosy. After excluding patients with incomplete data, 150 patients (107 in Lewoleba and 43 in Likupang) were included in the analysis. Mean ± standard deviation (SD) of the patients' age (years) was 44.71 ± 18.7, whilst median(s) ± range of the patients' length of disease (months) and length of symptoms before diagnosis (months) are 24 ± 720 and 6 ± 228, respectively. Majority of the patients were between 18-59 years old (64.67%), male (69.33%), graduated from elementary/primary schools (53.33%), did not have hand (73.33%) or foot (73.33%) disability, have no limitation nor restriction in functional activity (70.67%) and participation (88.67%), respectively, had been diagnosed with multibacillary type (82%), had none other family members living together diagnosed with leprosy (68.67%), have had leprosy for longer than 24 months (54.67%), and had been experiencing symptoms for more than six months before being diagnosed (48.67%) (**Table 2**).

Table 2. Demographics of Leprosy Patients in Lewoleba (Lembata) and Likupang (North Minahasa), Indonesia (n=150).

Characteristics	n	%

Age		
- ≤ 18	13	8.67
- 18-59	97	64.67
- ≥ 60	40	26.67
Gender		
- Female	46	30.67
- Male	104	69.33
Educational Background		
- Uneducated	9	6
- Primary Education	80	53.33
- Middle School	21	14
- Secondary Education	33	22
- Higer Education	7	4.67
WHO Hand Disability		
- Grade 0	114	73.33
- Grade 1	20	13.33
- Grade 2	14	9.33
WHO Foot Disability		

-	Grade 0	110	73.33
-	Grade 1	26	17.33
-	Grade 2	14	9.33
SALSA Score			
-	No limitation	106	70.67
-	Mild limitation	41	27.33
-	Moderate limitation	2	1.33
-	*Severe limitation*	*0*	*0*
-	Extreme limitation	1	0.67
Participation Scale			
-	No restriction	133	88.67
-	Mild restriction	10	6.67
-	Moderate restriction	6	4
-	*Severe restriction*	*0*	*0*
-	Extreme restriction	1	0.67
Jebsen-Taylor Hand Function Test (JTT)			
Dominant Hand			
-	< 20.94 s	75	50

- ≥ 20.94 s	75	50
Non-dominant Hand		
- < 24.88 s	75	50
- ≥ 24.88 s	75	50
Timed-up and go test		
- < 13.5 s	146	97.33
- ≥ 13.5 s	4	2.67
Location		
- Lembata	107	71.33
- Likupang	43	28.67
Number of Family Members with Leprosy Living Together		
- *None*	*103*	*68.67*
- 1	38	25.33
- 2	5	3.33
- *>2*	*3*	*2*
- Undisclosed	1	0.67
Types of Leprosy		
- Paucibacillary	27	18

- Multibacillary	123	82
Length of disease		
- < 24 months	63	42
- 24 months ++	82	54.67
- Undisclosed	5	3.33
Length of complaint before diagnosis		
- < 6 months	66	44
- 6 months ++	73	48.67
- Undisclosed	11	7.33

Analysis of the data was done according to ICF, introduced by the WHO in 2001. We divided the examinations according to several aspects. Age, gender, and types of leprosy are included into the patient's condition. The number of thickened nerves and WHO hand and foot disability grading represent body structure. Jebsen-Taylor Hand Function Test and TUG are parts of body function. The SALSA and Participation Scales represent activity and participation, respectively. Initially, we would like to find whether there are any differences in the patient's condition, body structure, body function, activity, and participation according to gender, age, education, and types of leprosy. All data were not normally distributed, and many sets of groups did not have equal variances. We found a significant difference in the number of thickened nerves between male and female (Mann-Whitney U test, median 0 vs 0, $p=0.0021$) as well as the number of nerve thickening occurrence in male compared to female (Fisher's exact test,

p=0.0041). Binary logistic regression showed OR of 3.626 (p=0.005) in males compared to female for the development of nerve thickening. However, adjusted OR showed 3.048 (p=0.099). There was also a significant difference in the frequency of multibacillary type infection in male and female (Fisher's exact test, p=0.0384) with OR of 2.532 (p=0.033) in males towards multibacillary type compared to females but adjusted OR of 2.018 (p=0.202). There were significant positive correlations between age and SALSA scales including the mobility (r=0.3965, p<0.0001) and dexterity (r=0.4738, p<0.0001) aspects, JTT in both hands, and TUG as seen in **Table 3**. Finally, we also found significant differences between levels of education in total SALSA scores, SALSA-Work scores, SALSA-Dexterity scores, JTT in both dominant and non-dominant hands, and TUG (**Figure 3A-E**).

Table 3. Significant bivariate analyses of age and other variables in ICF.

Other variables	Total SALSA	SALSA-Mobility	SALSA-Dexterity	JTT (dominant hand)	JTT (non-dominant hand)	TUG
Spearman's rho	0.5047	0.3965	0.4738	0.4923	0.4203	0.4904
p-value	<0.0001	<0.0001	<0.0001	<0.0001	<0.0001	<0.0001

SALSA, screening of activity limitation and safety awareness; JTT, Jebsen-Taylor hand function test; TUG, timed up and go test.

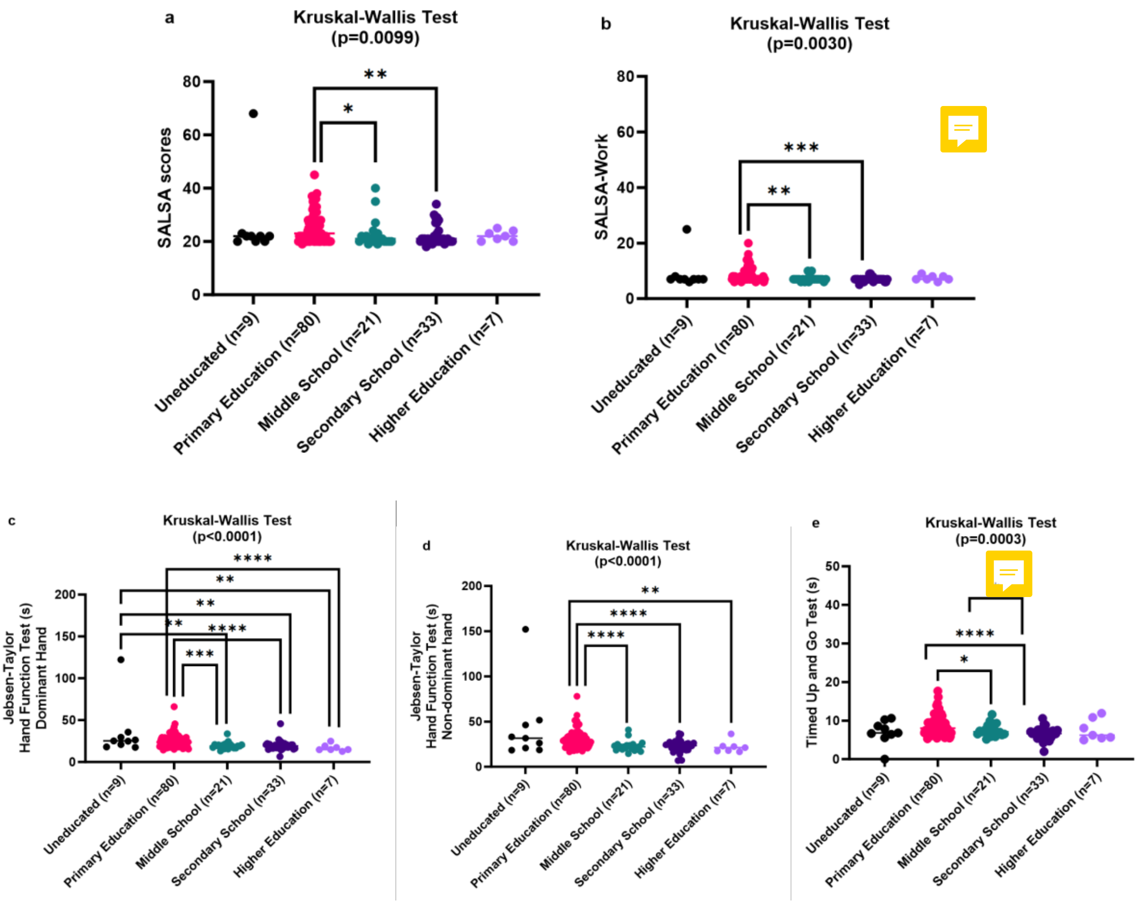

Figure 3. Comparison of SALSA and P-scales according to different educational levels (n=150). (A) Total SALSA scores. Mann-Whitney U tests showed statistically different scores between those who attended primary education vs middle school (median ± range = 23 ± 26 vs 21 ± 21, respectively, p=0.0127), as well as those who attended primary education and secondary school (median ± range = 23 ± 26 vs 21 ± 16, respectively, p=0.0025). (B) SALSA-Work scores. Mann-Whitney U tests yielded significant different scores between primary education vs middle school (median ± range = 7 ± 14 vs 7 ± 4, respectively, p=0.0092), as well as those who attended primary education and secondary school (median ± range = 7 ± 14 vs 7 ± 4, respectively, p=0.0003). (C) Jebsen-Taylor Hand Function Test in dominant hand. Significant differences were observed between uneducated (25.28 ± 104.6 s) vs middle school (19.06 ± 20.48 s, P=0.0091), secondary school (19.19 ± 39.19 s, p=0.0068), and higher education (14.97 ± 12.02 s, p=0.0033), as well as between primary education (23.71 ± 51.76

s) vs middle school (p=0.0002), secondary school (p<0.0001), and higher education (p=0.0004). (D) Jebsen-Taylor Hand Function Test in non-dominant hand. Mann Whitney U tests showed statistically significant differences between the group who only graduated from primary education (28.17 ± 61.40 s) and those who finished middle school (22.06 ± 26.31 s, p<0.0001), secondary school (23.51 ± 29.88 s, p<0.0001), and higher education (21.26 ± 19.82 s, p=0.0099). (E) Timed up and go test. Mann Whitney U tests showed significant differences between those who only attended primary education (8.015 ± 12.37 s) with those who attended middle school (7.110 ± 6.450 s, p=0.0348) and secondary school (6.650 ± 8.620 s, p<0.0001). Unpaired *t* test showed significant differences between those who attended middle school and secondary school (mean ± SD = 7.453 ± 1.516 s vs 6.585 ± 1.519 s, p=0.0454).

Next, we wanted to evaluate the environmental factors of ICF in our populations, so we divided the patients based on the areas of the community outreach; Likupang, North Sulawesi, and Lewoleba, West Nusa Tenggara. We found that there was a significant difference of mutibacillar leprosy occurrence between the population in Lewoleba and Likupang (Fisher's exact test, p=0.0017). Binary logistics regression analyses showed OR of 4.241 (p=0.001) for multibacillary occurrence in Lewoleba compared to Likupang. However, adjusted OR showed non-significant value of 1.843 (p=0.305). The occurrence of nerve enlargement between the participants living in Likupang and Lewoleba were found to seemingly different using Fisher's exact test (p<0.0001) but not with binary logistics (p=0.997). There were no significant differences of length of disease, length of symptoms before diagnosis, SALSA scores including mobility, work, and dexterity, P-scale, JTT in both hands, and TUG between Likupang and Lewoleba. Most of the patients did not have any people in the household who were also diagnosed with leprosy at the time of the examinations (**Table 2**). Chi-square tests between the number of household members with leprosy and the other variables showed no significant differences.

To assess different domains in relation to body structure impairment, Kruskal-Wallis tests were performed on SALSA scores, P-scales, JTT, and TUG according to WHO hand and foot disability grades. We found significant differences of the medians of SALSA scale, P-scale, and JTT in patients with grade 0, 1, and 2 WHO hand disability, whilst only comparison of SALSA scales in patients with grade 0, 1, and 2 WHO foot disability yields significant results as seen in **Figure 4 and 5**.

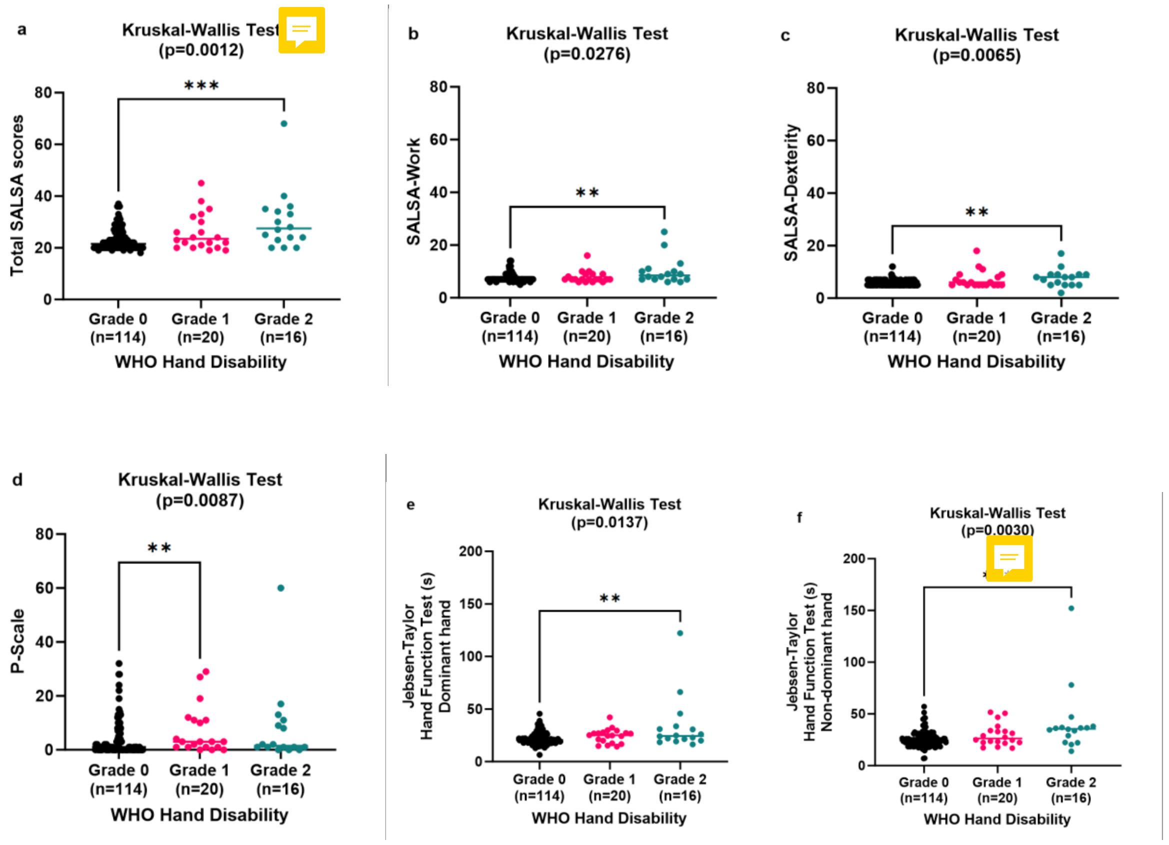

Figure 4. Comparison of hand structure impairment according to hand function impairment, activity limitation, and participation restriction (n=150). (A) Total SALSA scores. Mann-Whitney U test showed significant differences between grade 0 and grade 2 (median + range 21.50 + 19 vs 27.50 + 48, p=0.0004). (B) SALSA-Work. Mann-Whitney U test showed significant differences between grade 0 and grade 2 (7 + 9 vs 8.5 + 19, p=0.0058). (C) SALSA-Dexterity. Mann-Whitney U test showed significant differences between grade 0 and grade 2 (5 + 7 vs 8 + 15, p=0.0018). (D) P-Scale. Mann-Whitney U test showed significant

differences between grade 0 and grade 1 (1 ± 32 vs 3 ± 29, p=0.0018). (E) JTT dominant hand. Mann-Whitney U test showed significant differences between grade 0 and grade 2 (20.5 ± 39.06 s vs 24.51 ± 105.8 s, p=0.0077). (F) JTT non-dominant hand. Mann-Whitney U test showed significant differences between grade 0 and grade 2 (24.37 ± 50.03 s vs 35.62 ± 138.2 s, p=0.0009).

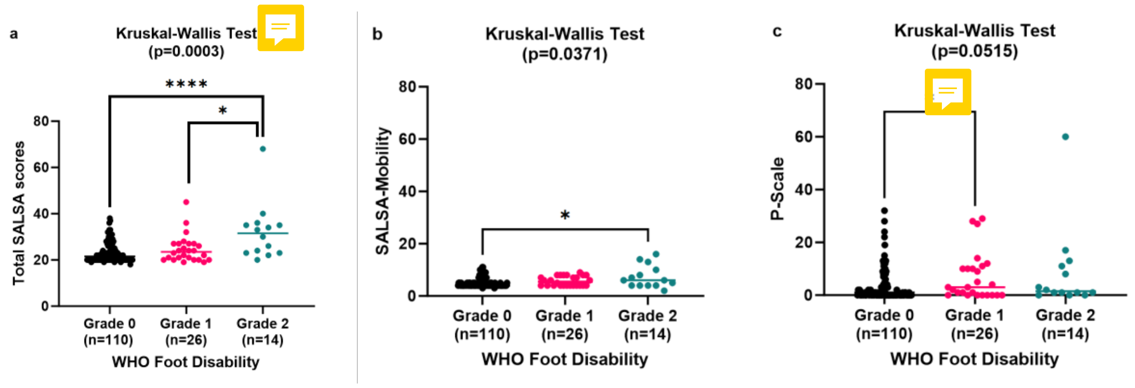

Figure 5. Comparisons of foot structure impairment according activity limitation and participation restriction (n=150). (A) Total SALSA scores. Mann-Whitney U test showed significant differences between grade 0 and grade 2 (median ± range 21.25 ± 20 vs 31.50 ± 48, p<0.0001) and between grade 1 and grade 2 (23.5 ± 26 vs 31.50 ± 48, p=0.0146). (B) SALSA-Mobility. Mann-Whitney U test showed significant differences between grade 0 and grade 2 (4 ± 8 vs 6 ± 14, p=0.0339). (C) P-scale. Mann-Whitney U test showed significant differences between grade 0 and grade 2 (1 ± 32 vs 4 ± 29, p=0.0413).

Next, we wanted to see if the type of leprosy affected the other variables from the other domains of ICF. Comparison tests of body structure impairment, body function impairment, activity limitation, and participation restriction between types of leprosy; paucibacillary or multibacillary, were performed. We found no significant differences. However, Fisher's exact tests showed that the occurrence of nerve enlargements were significantly higher in multi-bacillary cases (p=0.0024). Binary logistic regression analyses showed OR of 7.468 (p=0.008)

and adjusted OR of 29.782 (p=0.015) for occurrence of nerve enlargement in multibacillary type compared to paucibacillary leprosy. Bivariate analysis showed that the length of disease, length of symptoms before diagnosis, and number of nerve thickenings were found to be independent to all other variables.

Finally, we wanted to see whether the variables in the body function domain were inter-correlated with the activity limitation and participation restriction domains in the study population (**Figure 6**). There were positive correlations within the total SALSA-scores and SALSA-Mobility, -Work, and -Dexterity. There was a positive and significant correlation between P-scale and SALSA-mobility scores. There were positive and significant correlations between total SALSA scores, SALSA-work, and -dexterity and the Jebsen-Taylor hand function test. We also found significant correlations between TUG and SALSA along with SALSA-mobility scores. There were positive and significant correlations between JTT in dominant and non-dominant hands, TUG and SALSA-Work, and TUG and JTT in both hands.

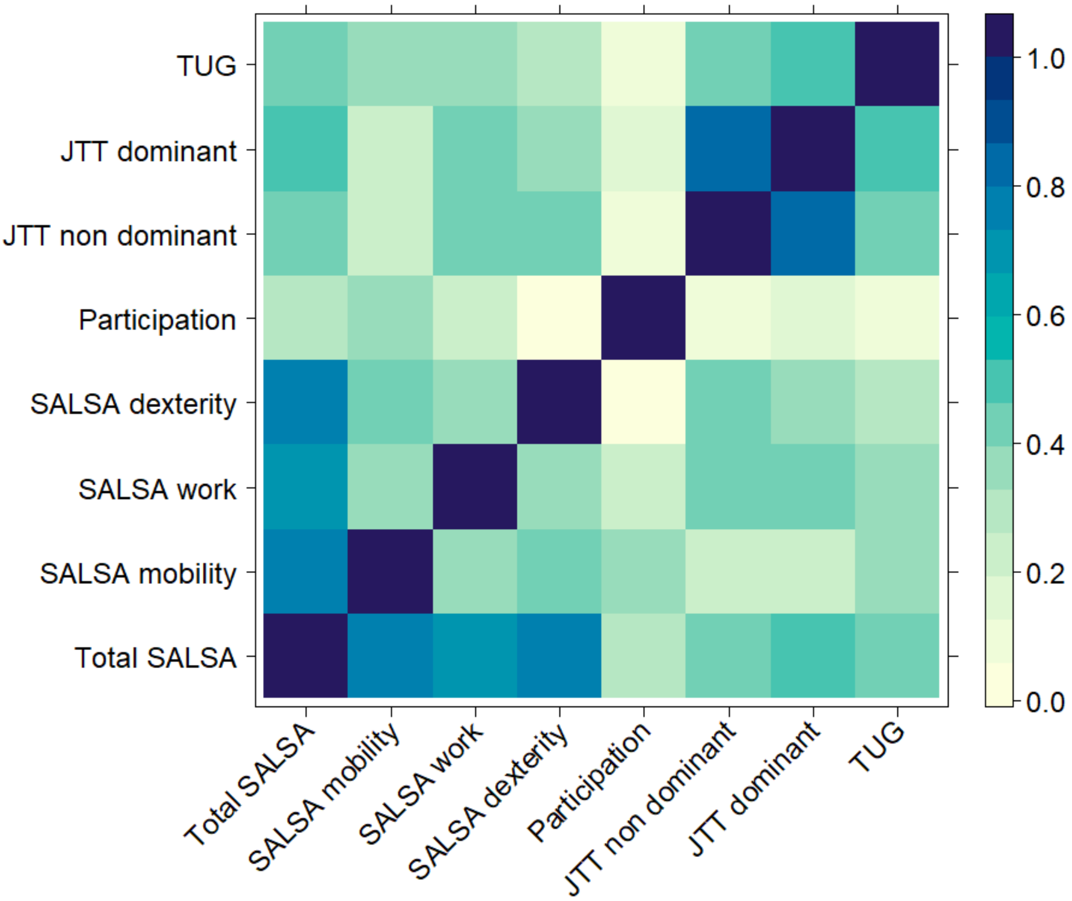

Figure 6. Bivariate analyses of the variables in the body function, activity limitation, and participation restriction domains according to the International Classification of Functioning, Disability and Health (ICF) (n=150). Participation restriction (P-scale) and SALSA-work (Spearman's rho=0.3347, p<0.0001)), body function impairment (TUG) and activity limitation in relation to lower extremities (total SALSA (Spearman's rho=0.4232, p<0.0001) and SALSA-mobility (Spearman's rho=0.3296, p<0.0001)), body function impairment (JTT) and activity limitation (total SALSA (Spearman's rho=0.4659, p<0.0001 and Spearman's rho=04296, p<0.0001), SALSA-work (Spearman's rho =0.4236, p<0.0001 and Spearman's rho=0.3967, p<0.0001), and SALSA-dexterity (Spearman's rho=0.3737, p<0.0001 and Spearman's rho=0.4212, p<0.0001) related to upper extremities in dominant and non-

dominant hands, respectively. SALSA, screening of activity limitation and safety awareness; JTT, Jebsen-Taylor hand function test; TUG, timed up and go test.

Discussion

One of the purposes of this study is to utilize ICF to assess the functional disabilities experienced by individuals diagnosed with leprosy in Likupang, North Minahasa and Lewoleba, Lembata, Indonesia. ICF recognizes several dimensions of disability, including impairment in body structure and function, restrictions in activity, and limitations in participation. Additionally, the classification acknowledges the significant influence of physical and social environmental factors on the outcomes of disability. In this study, we found that hand and foot disability affected the activity limitation and participation restriction domains in this population. Hand disability also affected body function domains. Both participation restriction and activity limitation had significant correlation to one another. However, activity limitations also had significant correlation with upper and lower body functions. Activity limitation and body functions were affected by age and educational levels. We also found that locations of the population and the types of leprosy did not significantly affect disability progression. All in all, our results imply that the body structure and body function domain in ICF affected activity limitation and participation restriction and vice versa in leprosy patients [4].

Several personal factors for leprosy and leprosy-related disabilities have been studied. A meta-analysis of 17 studies found that leprosy was associated with male gender, performing manual labour, ever food-shortage, sharing household with another leprosy patient, and communal living with a minimum of 5 occupants [36]. Moreover, risk factors for leprosy-related disabilities include advanced age, male, low education, multibacillary leprosy type, the

presence of leprosy reactions, nerve damage, delayed diagnosis, and treatment dropout [37-42].

Findings related to demographic factors in this study, which seemed to affect the domains of ICF disability, have been observed in other studies. Most of the patients in this study were males within the range of productive age group, as also found in other studies from Indonesia, Brazil, and India [43-46]. Our results also showed that body function, activity limitation, and participation restriction had positive correlations with age. Higher male population in leprosy cases could be attributed to differences in sociocultural behaviours between genders and life choices that would lead to higher risk of contracting the infection [47]. More than half of this study population also had low educational attainment, indicating a shared context of socioeconomic vulnerability. This trend has been observed in Indonesian, Indian, and Brazilian studies by Menaldi et al.[8], Karotia et al. [48], and Dergan et al. [46], respectively. Unfortunately, diagnostic timepoints and treatment dropout were not explored.

Leprosy patients with multi-bacillary type demonstrate more pronounced physical dysfunction and experience greater bodily pain when compared to the pauci-bacillary group, which primarily stems from nerve impairment in leprosy patients [49]. Multi-bacillary patients were significantly at higher risk of developing nerve-function impairment [50]. Additionally, a study by Lustosa et al. (2011) revealed that multibacillary patients were associated with lower SF-36 scores across four domains: physical functioning, bodily pain, general health perceptions, and social functioning. These patients were also at higher risk of being found with grade II disability at the time of diagnosis [51].

Increased nerve thickening has been found to play a role in delaying diagnosis and potentially serves as a risk factor for deformity development [52]. Deformities were also more often seen in those with more than 3 thickened nerves. Our bivariate analyses found that the number of

nerve enlargement, functional activity limitation and social participation restriction in leprosy patients are independent with each other, which was represented by SALSA scale (r=0.165, p=0.044) and P-scale (r=0.167, p=0.041), respectively. In accordance with Hinkel's criteria, when the correlation coefficient falls below 0.30, it signifies a minimal or negligible association between the two variables [35]. It is worth noting that previous research has indicated that abnormalities and neuropathic pain can manifest even in the absence of nerve enlargement [53, 54]. Consequently, functional activity limitations and social participation restrictions may persist even in the absence of observable nerve enlargement.

There were 29.33% of participants who experienced mild (27.33%), moderate (1.33%), and extreme (0.67%) activity limitations. This proportion is lower than that reported by Shivanna et al. [55] in India, Nascimento et al. [56] in Brazil, and Abdela et al. [57] in Ethiopia. In two studies (Shivanna et al. [55] and Nascimento et al. [56]), most participants only felt mild functional limitation. High proportion of patients with activity limitations may indicate prior delayed diagnosis, inadequate management, and lack of physical rehabilitation service [55-57].

Patients in this study who had limitations in social participation accounted for only one-eighth of the total participants, with 6.67% experiencing mild restriction, 4% experiencing moderate restriction, and 0.67% experiencing extreme restriction. These figures are notably lower than those found in studies conducted in Brazil, Ethiopia, and Nigeria, which reported participation restriction rates of 24%, 55.1%, and 89.3%, respectively [56-58]. Variation in cultural characteristics, socioeconomic conditions, stigma, and availability of rehabilitative services may account for these differences [56].

Functional limitation and societal restriction are impacted by many factors. According to a previous study by Nogueira in Fortaleza, most of elderly people with leprosy can still do basic

daily tasks such as opening bottles with screw caps, cooking, carrying heavy objects, and walking uneven ground [59]. Stigma and prejudice associated with leprosy can lead to societal exclusion even in the absence of visible lesions or impairments [56]. Several factors such as self-stigmatization, activity limitations, family-related issues, poverty, low education levels, inadequate rehabilitation services that include community-based rehabilitation programs and community ignorance about the disease and its transmission can affect societal participation restriction [57].

This study also found significant differences of SALSA scores according to the degree of WHO disability for hands and feet (p=0.0012 and p=0.0003, respectively). Similar findings from de Souza et al. (2016) indicated that SALSA scores are associated with the degree of impairment ($p<0.01$) [30].

There were significant differences between total participation scale and hand disability based on WHO disability grading (p=0.0087). Grade 2 disabilities, particularly those affecting the hands and especially with deformities and resulting amputations can cause discomfort and limit patients' ability to interact with their environments, participate in community activities, and pursue employment opportunities. This may lead to reduced autonomy outside the home and interference with family roles [60].

Bivariate analyses also found significant positive correlations between JTT in both dominant and non-dominant hands, and SALSA scores, including the work and dexterity domains, suggesting that reduced hand function is paralleled with limited functional activity. Meanwhile, TUG test was used to determine functional mobility, which is typically reduced in people with foot disability [61]. There were positive correlations between TUG test and both SALSA and mobility domain of SALSA scale. TUG test and total participation scale denoted no significant

correlation with p=0.3956. There were also no significant differences of total participation scale between different WHO foot disability grades (p=0.0515).

We found an interesting trend where hand function affected participation restriction whereas foot did not. In an older study of 63 patients with hereditary motor and sensory neuropathy (HMSN) or Charcot-Marie-Tooth disease (CMT), upper limb disability, and not lower extremity, was considered to correlate independently with participation restriction [62]. The latter has been linked to mortality and morbidity with age. We observe a similar trend of association between the hand function and societal participation in a recent study by Akbarfahimi et al. (2021) among 84 stroke patients [63]. Upper limb function measured using Fugl-Meyer scale was found to correlate significantly with participation score (r = 0.315, p =0.003). In an older study with 2291 healthy participants above 50 years of age, lower extremity strength and balance were not significantly associated with societal participation [64]. However, the odds of having self-reported limitation in social participation were higher in those with slower gait speed or less than 1 m/s (odds ratio [OR] = 3.1 99% confidence interval [CI] 1.5–6.2) compared to those above. Some studies have even shown supporting evidence of a relationship between gait speed and longer life. However, none of the studies discussed the reason behind this trend. Future studies detailing analysis of upper and lower extremity functions and disabilities are needed.

Most of the population in Likupang work as fishermen, which may be the cause of higher participation rates compared to studies in other countries [65,66]. Since fisherman's work is rather self-reliant, they are not really affected by the stigma around them. However, fishermen required fully functioning hands to maintain their job. Most of them use handline fishing, using hand grip and strength to be used. In addition to fishing, people of Likupang also work in other industries as well, such as farming and construction workers [67, 68]. The main occupation in Lembata island is corn farming industry and most of the farmers spent around 60% of their

working time on the field [67,69]. Working in this industry demands people to have high functioning hands, due to its labor characterization of this job [70]. Farmers and fishermen with leprosy will suffer greatly mainly because of their incapability to work and use their tools properly. This study showed a significant correlation between hand disability and participation scale, which include occupational activities such as fishing and farming.

Based on the ICF model, environmental and personal factors are also important components of disability. Risk factors for leprosy in Indonesia include floor height, house ventilation area, house window usage habits, floor and wall types, environmental sanitation, history of household contact, residential density, humidity, and economic status [71-73]. Humidity poses a significantly increased risk, with an 8.415-fold greater likelihood, of leprosy occurrence within the community. Similarly, personal hygiene presents a 6.926-fold higher risk of leprosy occurrence in the community. Residential density in Indonesian society is associated with a substantially elevated risk, estimated to be 5.754 times greater, of experiencing leprosy [73].

The populations in Lewoleba and Likupang were unequally distributed with several areas exhibiting higher density than others. There were also inadequate access to clean water, insufficient drainage and sanitation systems, and relatively high levels of humidity, all of which are risk factors for leprosy as well as posing further challenges in managing leprosy [74, 75]. One possible approach to preventing leprosy in remote and rural areas is by providing public education on maintaining the physical condition of houses in accordance with established standards. This includes ensuring the presence of ceilings, utilizing easily cleanable flooring materials, maintaining comfortable humidity levels, and limiting the occupancy of bedrooms to a maximum of two individuals [76]. Conversely, marital status and proximity to healthcare facilities do not significantly affect the likelihood of leprosy-related disability [39].

Limitations

This study has several limitations. Firstly, this study only included the patients who were willing to participate and excluded those who would potentially be more reclusive and isolated. Secondly, the population included in the study also came from two different areas, each with unique social and environmental characteristics. There may be some gaps in demographical factors, which we could not currently analyze, that would influence the outcome of the study. Finally, although we found that the personal factors identified in our study such as age, gender, and educational background significantly contribute to leprosy-related disability, other influential factors including personal hygiene, contact history, socio-economic status, health beliefs, and religion were not specifically examined in our research [72,77-79], highlighting the need for further exploration and investigation.

Conclusion

Through this study, we proved that the ICF can be used to identify factors relating to leprosy patients' disability progression in Indonesia. We found several personal factors such as age and education as upstream factors of disability development in leprosy patients through their actions towards activity limitation and body functions. In addition, participation restriction also affected the development of disability. We recommend clinicians to address all these factors to adjust their treatment approach as a preventive measure for the development of disability in leprosy patients. This study also emphasizes on the importance of equal access for primary and secondary education in Indonesia, especially in remote areas. Future studies related to the impact of education intervention in rural populations towards disease and disability preventions should be done.

Acknowledgment

This work is supported by the Directorate of Research and Community Engagement of Universitas Indonesia. We would like to thank the directors and staffs of St. Damian Hospital Lewoleba-Lembata-East Nusa Tenggara, Ministry of Health East Lewoleba-East Nusa Tenggara, Ministry of Health North Minahasa-North Sulawesi, and Faculty of Medicine, University of Sam Ratulangi, Manado City-North Sulawesi. We would like to acknowledge the KATAMATAKU team and the medical students of Universitas Indonesia.

References

1. Lastoria JC, de Abreu MAMM. Leprosy: review of the epidemiological, clinical, and etiopathogenic aspects - Part 1. An Bras Dermatil. 2014;89(2): 205-218. doi: 10.1590/abd1806-4841.20142450.
2. Rathod SP, Jagati A, Chowdhary P. Disabilities in leprosy: an open, retrospective analyses of institutional records. An Bras Dermatol. 2020;95(1): 52-56. doi: 10.1016/j.abd.2019.07.001.
3. Mowla MR, Angkur DM, Hasan Z, Sultana MN, Afrin S, Akhter MS. Leprosy patients with deformities at post- elimination stage: The Bangladesh experience. Skin Health Dis. 2021;1(1): e5. doi: 10.1002%2Fski2.5.
4. van Brakel WH, Sihombing B, Djarir H, Beise K, Kusumawardhani L, Yulihane R, et al. Disability in people affected by leprosy: the role of impairment, activity, social participation, stigma and discrimination. Glob Health Action. 2012; 5. doi: 10.3402/gha.v5i0.18394.
5. Babik I, Gardner ES. Factors affecting the perception of disability: A developmental perspective. Front Psychol. 2021;12. doi: 10.3389/fpsyg.2021.702166.
6. Control of Neglected Tropical Diseases (WHO). Global leprosy (Hansen disease) update, 2021: moving towards interruption of transmission. WHO; 2022. WER No.: 36(97); 429–450.
7. Widi S. Prevalensi kusta di Indonesia meningkat pada 2022. DataIndonesia.id. 2023 Jan 31 [cited 2023 Apr 14]. Available from: https://dataindonesia.id/ragam/detail/prevalensi-kusta-di-indonesia-meningkat-pada-2022.
8. Menaldi SL, Harini M, Nelfidayani N, Irawati Y, Setiono S, Wahyuni LK, et al. Functional activity limitation of leprosy cases in an endemic area in Indonesia and

recommendations for integrated participation program in society. PLoS Negl Trop Dis. 2022;16(8): e0010646. doi: 10.1371/journal.pntd.0010646.

9. Gunawan H, Kamilia A, Menaldi SL, Marissa M, Prakoeswa CR, Alinda MD, et al. Characteristics of grade 2 disability in indonesian children with leprosy: A five-year multicenter retrospective study. Clin Cosmet Investig Dermatol. 2021;14: 1149-53. doi: 10.2147/CCID.S325858.
10. Direktorat Pencegahan dan Pengendalian Penyakit Menular. Laporan kinerja 2022. Kementrian Kesehatan Republik Indonesia. 2023 [cited 2023 Jul 07]. Available from: https://p2pm.kemkes.go.id/storage/informasi-publik/content/GHwE3BiLbOrvZZPKY1Pm91BIRWqzE4-metaTGFwa2luIFAyUE0gMjAyMi5wZGY=-.pdf.
11. Control of Neglected Tropical Diseases, SEARO Regional Office for the South East Asia (RGO), ed. Cooreman EA. Towards zero leprosy: Global leprosy (Hansen's disease) strategy 2021-2030. WHO. 2021 [cited 2023 Jul 07]. Available from: https://www.who.int/publications/i/item/9789290228509.
12. Ogunsumi DO, Lal V, Puchner KP, van Brakel W, Schwienhorst-Stich E-M, Kasang C, et al. Measuring endemicity and burden of leprosy across countries and regions: A systematic review and Delphi survey. PLoS Negl Trop Dis. 2021; 15(9): e0009769. doi: 10.1371/journal.pntd.0009769.
13. World Health Organization: Control of Neglected Tropical Diseases, ed. Cooreman EA. Guidelines for the diagnosis, treatment and prevention of Leprosy [Internet]. 2018 [cited 2023 September 10]. Available from: https://www.who.int/publications/i/item/9789290226383.

14. Badan Meteorologi, Klimatologi, dan Geofisika. Kelembaban menurut kabupaten/kota (persen), 2020-2022 [cited 2023 Jul 5]. Available from: https://ntt.bps.go.id/indicator/151/959/1/kelembaban-menurut-kabupaten-kota.html.
15. Badan Pusat Statistik Kabupaten Lembata ed. Kabupaten Lembata dalam angka 2022. Badan Pusat Statistik Kabupaten Lembata; 2022. Catalog No.: 1102001.5308. Publication No.: 53080.2202.
16. Pakasi TT. Laporan validasi data kusta tahun 2021. Kemenkes RI; 2021. Report No.: 2388.
17. Siburian CE, Wicaksono P, Perdata IRD. Kecamatan Likupang Timur dalam angka. BPS-Statistics of Minahasa Utara Regency. 2022 Sept [cited 2023 Jun 10]. Available from: https://minutkab.bps.go.id/publication/2022/09/26/aed2294e0e44f1a000c218fe/kecamatan-likupang-timur-dalam-angka-2022.html.
18. Siburian CE, Wicaksono P, Perdata IRD. Kecamatan Likupang Barat dalam angka. BPS-Statistics of Minahasa Utara Regency. 2022 [cited 2023 Jun 10]. Available from: https://minutkab.bps.go.id/publication/2022/09/26/ed30d43110989d0bdd02deff/kecamatan-likupang-barat-dalam-angka-2022.html.
19. Siburian CE, Wicaksono P, Perdata IRD. Kecamatan Likupang Selatan dalam angka. BPS-Statistics of Minahasa Utara Regency. 2022 [cited 2023 Jun 10]. Available from: https://minutkab.bps.go.id/publication/2022/09/26/c6e9b24b7fcd3b642533311a/kecamatan-likupang-selatan-dalam-angka-2022.html.
20. Badan Meteorologi dan Geofisika. Prakiraan cuaca Kabupaten Minahasa Utara – Provinsi Sulawesi Utara. BMKG. 2023 [cited 2023 Jun 10]. Available from: https://www.bmkg.go.id/cuaca/prakiraan-cuaca.bmkg?AreaID=501530&Prov=31&lang=ID.

21. Badan Pusat Statistik Kabupaten Minahasa Utara. Penduduk, laju pertumbuhan penduduk, distribusi persentase penduduk, kepadatan penduduk, rasio jenis kelamin penduduk menurut kecamatan di Kabupaten Minahasa Utara, 2022. BPS- Kabupaten Minahasa Utara; 2023 [cited 2023 Jun 10]. Available from: https://minutkab.bps.go.id/statictable/2023/03/09/154/penduduk-laju-pertumbuhan-penduduk-distribusi-persentase-penduduk-kepadatan-penduduk-rasio-jenis-kelamin-penduduk-menurut-kecamatan-di-kabupaten-minahasa-utara-2022.html.
22. Kementrian Kesehatan Indonesia. Laporan validasi data kusta tahun 2021. Kementerian Kesehatan Republik Indonesia. 2022 [cited 2023 Jun 11]. Available from: https://p2pm.kemkes.go.id/storage/informasi-publik/content/informasi-publik_18_20220718073519.pdf.
23. Budiawan T, Ferdiana A, Daendel S, Widayati R, de Hart J, Soesman M, et al. "We are not afraid anymore…" Capturing the most significant change of the Leprosy Friendly Village approach in North Sulawesi, Indonesia. Leprosy Review. 2020; 91(2):173-189. doi: 10.47276/lr.91.2.173.
24. Serdar CC, Cihan M, Yücel D, Serdar MA. Sample size, power and effect size revisited: simplified and practical approaches in pre-clinical, clinical and laboratory studies. Biochem Med (Zagreb). 2021 Feb 15;31(1):010502. doi: 10.11613/BM.2021.010502.
25. The SALSA Collaborative Study Group. SALSA scale (Screening Activity Limitation and Safety Awareness) users manual version 1.1 [Internet]. Netherlands; 2010 [cited 2023 September 20]. Available from: https://www.infontd.org/toolkits/nmd-toolkit/salsa-scale.
26. The Participation Scale Development Team. Participation scale users manual version 6.0 [Internet]. Netherlands; 2010 [cited 2023 September 20]. Available from:

https://www.infontd.org/toolkits/ntd-morbidity-and-disability-toolkit/participation-scale-p-scale.

27. Akkerman L. The quantitative cultural validation of three tools among people affected by Leprosy in West Java, Indonesia. VU University Amsterdam. 2016:53. Available from: https://www.infontd.org/resource/quantitative-cultural-validation-three-tools-among-people-affected-leprosy-west-java.

28. Kelders R, van Brakel W, Beise K, Irwanto. Testing and validating a simplified scale to measure social participation of people with disabilities in Indonesia. Disabil Rehabil. 2012;34(8):638-46. doi: 10.3109/09638288.2011.615369.

29. World Health Organization. International classification of functioning, disability and health. 2001 [cited 2023 Apr 14]. Available from: https://apps.who.int/iris/bitstream/handle/10665/42407/9241545429.pdf?sequence=1.

30. De Souza VT, Júnior WM, De Jesus AM, De Oliveira DT, Raptis HA, De Freitas PH, et al. Is the WHO disability grading system for leprosy related to the level of functional activity and social participation? Leprosy Review. 2016;87(2):191-200. doi: 10.47276/lr.87.2.191.

31. Sigirtmac IC, Oksuz C. Investigation of reliability, validity, and cutoff value of the Jebsen-Taylor Hand Function Test. Journal of Hand Therapy. 2020: 1-7. doi: 10.1016/j.jht.2020.01.004.

32. Bohannon RW. Reference values for the Timed Up and Go Test: a descriptive meta-analysis. J Geriatr Phys Ther. 2006;29(2):64-68.

33. The Salsa Collaborative Study Group, The Salsa Collaborative Study Group. The development of a short questionnaire for screening of activity limitation and safety awareness (SALSA) in clients affected by leprosy or diabetes. Disabil Rehabil. 2007;29(9): 689–700. pmid:17453991.

34. Souza MA, Coster WJ, Mancini MC, Dutra FC, Kramer J, Sampaio RF. Rasch Analysis of the Participation Scale (P-scale): Usefulness of the P-scale to a rehabilitation services network. BMC Public Health. 2017;17(934). doi: 10.1186/s12889-017-4945-9.
35. Hinkle DE, Wiersma W, Jurs SG. Applied statistics for the behavioral sciences. 5th ed. Belmont: Wadsworth. 2003.
36. Pescarini JM, Strina A, Nery JS, Skalinski LM, Andrade KVFD, Penna MLF, et al. Socioeconomic risk markers of leprosy in high-burden countries: A systematic review and meta-analysis. PLoS Negl Trop Dis. 2018;12(7): e0006622. doi:10.1371/journal.pntd.0006622.
37. Wardhani RK, Wahyuni LK, Harini M, Lakmudin A. Hand disability grading in leprosy: Associated Risk Factors. Indian J Lepr. 2020;92:183-189.
38. de Paula HL, de Souza CDF, Silva SR. risk factors for physical disability in patients with leprosy: A systematic review and meta-analysis. JAMA Dermatol. 2019;155(10):1120-1128. doi:10.1001/jamadermatol.2019.1768.
39. Cisneros J, Ferreira JA, de Faria Grossi MA, de Filippis T, de Oliveira AL, Lyon S, et al. Associations between occupation, leprosy disability and other sociodemographic factors in an endemic area of Brazil. PLOS Global Public Health. 2022; 2(9): e0000276. doi: 10.1371/journal.pgph.0000276.
40. Chen X, Liu H, Shui T, Zha S. Risk factors for physical disability in patients with leprosy disease in Yunnan, China: Evidence from a retrospective observational study. PLoS Negl Trop Dis. 2021;15(11): e0009923. Doi: 10.1371/journal.pntd.0009923.
41. Srinivas G, Muthuvel T, Lal V, Vaikundanathan K, Schwienhorst-Stich EM, Kasang C. Risk of disability among adult leprosy cases and determinants of delay in diagnosis in five states of India: A case-control study. PLoS Negl Trop Dis. 2019;13(6):e0007495. doi: 10.1371/journal.pntd.0007495.

42. Veras GCB, Junior JFL, Candido EL, Maia ER. Risk factors for physical disability due to leprosy: a case-control study. Cad. Saude Colet. 2021;29(3). doi:10.1590/1414-462X202129030182.
43. Lubis RD, Darmi M, Prakoeswa CR, Agusni RI, Kusumaputra BH, Alinda MD, et al. Leprosy epidemiology according to leprosy type in 13 teaching hospitals in Indonesia between 2018 and 2020. Open Access Macedonian Journal of Medical Sciences. 2022;10(E):1812-17. Doi: 10.3889/oamjms.2022.10816.
44. Arif T, Amin SS, Adil M, Dorjay K, Raj D. Leprosy in the post-elimination era: a clinico-epidemiological study from a northern Indian tertiary care hospital. Acta Dermatovenerologica. 2019;28:7-10. Doi: 10.15570/actaapa.2019.2.
45. Nery JS, Ramond A, Pescarini JM, Alves A, Strina A, Ichihara MY, et al. Socioeconomic determinants of leprosy new case detection in the 100 million Brazilian Cohort: a population-based linkage study. The Lancet Global Health. 2019;7(9):e1226-e1236. Doi: 10.1016/S2214-109X(19)30260-8.
46. Dergan MR, Gonçalves NV, Falcão LF, Carvalho DD, Anjos TA, Palácios VR. Socio-demographic and epidemiological characterisation of leprosy in the population of Pará, Amazon, from 2016 to 2020. Archives of Current Research International. 2023;23(5): 34-39. doi: 10.9734/ACRI/2023/v23i5573.
47. Costa RD, Mendonça VA, Penido RA, Lyon S, Costa AM, Costa MD, et al. Study of the profile of the neutrophin BDNF in new leprosy cases before, during and after multidrug therapy. Arq Neuropsiquiatr. 2011;69:100-104.
48. Karotia D, Kishore J, Kumar A. Epidemiological determinants of leprosy in a high endemic district of India: A community based case control study. Indian J Lepr. 2022;94: 69-80.

49. Albuquerque RG, Buratto GG, Hirotsu C, Maeda SM, Floriano MC, Andersen ML, et al. Comparison of quality of life evaluated by SF-36 and DLQI in multibacillary and paucibacillary leprosy patients from Sao Paulo, Brazil. International Journal of Dermatology. 2019;58(12):1415-22. Doi: 10.1111/ijd.14489.
50. van Brakel WH, Nicholls PG, Wilder-Smith EP, Das L, Barkataki P, Lockwood DNJ, et al. Diagnosis of neuropathy in leprosy—comparing diagnostic tests in a large prospective study (the INFIR Cohort Study). PLOS Neglected Tropical Disease. 2008;2(4):e212. Doi: 10.1371/journal.pntd.0000212
51. Lustosa AA, Nogueira LT, Pedrosa JI, Teles JB, Campelo V. The impact of leprosy on health-related quality of life. Rev. Soc. Bras. Med. Trop. 2011;44(5). Doi: 10.1590/S0037-86822011000500019.
52. Kumar A, Girdhar A, Girdhar BK. Nerve thickening in leprosy patients and risk of paralytic deformities: a field based study in Agra, India. Lepr Rev. 2004;75: 135-142.
53. Virmond M, Grzybowski A, Virmond L. Leprosy: A glossary. Clinics in dermatology. 2015 Jan 1;33(1):8-18.
54. Garbino JA, Heise CO, Marques Jr W. Assessing nerves in leprosy. Clinics in dermatology. 2016 Jan 1;34(1):51-8.
55. Shivanna PH, Shruthi RT. Profile of functional limitation using Screening of Activity Limitation and Safety Awareness Scale in leprosy population of Mysore district. Physiotherapy-The Journal of Indian Association of Physiotherapists. 2022;16(2): 81-83. Doi: 10.4103/pjiap.pjiap_17_22.
56. Nascimento DD, Ramos Jr AN, Araújo OD, Macêdo SF, Silva GV, Lopes WM, et al. Activity limitation and social participation restriction of people with leprosy: a cross-sectional analysis of magnitude and associated factors in a hyperendemic municipality

in the state of Piauí, Brazil, 2001-2014. Epidemiologia e Serviços de Saúde. 2020;29(3). Doi: 10.5123/S1679-49742020000300012.

57. Abdela SG, van Henten S, Abegaz SH, Bayuh FB, Zewdu FT, Berhe FT, et al. Activity limitation and social participation restriction among leprosy patients in Boru Meda Hospital, Amhara Region, Ethiopia. PLoS Neglected Tropical Disease. 2020;14(9). Doi: 10.1371/journal.pntd.0008702.
58. Dahiru T, Iliyasu Z, Aliyu MH. Social participation restriction among persons with leprosy discharged from a multidrug therapy clinic in northern Nigeria. Transactions of The Royal Society of Tropical Medicine and Hygiene. 2022;116(8): 694-703. Doi: 10.1093/trstmh/trac056.
59. Nogueira PS, Barbosa RG, Almeida PC, Florêncio CM, Marques MB, Teles LM. Applicability of the instrument "Screening of Activity Limitation and Safety Awareness" in elderly with leprosy. Esc Anna Nery. 2020;24(2): e20190251.
60. Arif T, Solikhah FK. Disability in Leprosy: Daily activities and social participation in Indonesia. Ethno Med. 2021;15(3-4): 168-174. Doi: 10.31901/24566772.2021/15.3-4.640.
61. Gaino JZ, Bértolo MB, Nunes CS, Sachetto Z, Landim SF, de Paiva Magalhães E. The Structural Index Score and its relation to foot function, disability and physical performance tests in rheumatoid arthritis (RA) — A cross-sectional study. The Foot. 2022;51. Doi: 10.1016/j.foot.2021.101876.
62. Videler A, Beelen A, van Schaik I, de Visser M, Nollet F. Limited upper limb functioning has impact on restrictions in participation and autonomy of patients with hereditary motor and sensory neuropathy 1a. Journal of Rehabilitation Medicine. 2009;41(9): 746–750. doi:10.2340/16501977-0419.

63. Akbarfahimi N, Mazidi MH, Hosseini SA, Vahedi M, Amirzargar N. The relationship between upper limb function and participation and independence in daily activities of life in people with stroke. Journal of Rehabilitation. 2021;22(1):86-101. doi: 10.32598/RJ.22.1.3238.1.

64. Warren M, Ganley KJ, Pohl PS. The association between social participation and lower extremity muscle strength, balance, and gait speed in US adults. Prev Med Rep. 2016 Jun 7;4:142-7. doi: 10.1016/j.pmedr.2016.06.005.

65. Budianto PF, Susilo E, Indrayani E. Implementasi pengembangan pariwisata di pulau-pulau kecil terhadap masyarakat pesisir Desa Lihunu, Kecamatan Likupang, Kabupaten Minahasa Utara, Provinsi Sulawesi Utara. Jurnal ECSOFiM. 2013;1(1).

66. Mamahit OO, Andaki JA, Dien CR, Jusuf N, Tambani GO, Lantu S. Persepsi anak terhadap mata pencaharian nelayan di Desa Bahoi Kecamatan Likupang Barat Kabupaten Minahasa Utara. Akulturasi. 2022;10(1).

67. Badan Pusat Statistik Kabupaten Lembata ed. Kabupaten Lembata dalam Angka 2018. Badan Pusat Statistik Kabupaten Lembata. 2018.

68. Liando VY, Aling DRR, Wasak MP. Strategi adaptasi sosial dan ekonomi nelayan alat tangkap Sero di Desa Jayakarsa Kecamatan Likupang Barat Kabupaten Minahasa Utara. AKULTURASI: Jurnal Ilmiah Agrobisnis Perikanan. 2020;8(1):62-66

69. Bupati Lembata Nusa Tenggara Timur. Penetapan Kawasan Pertanian Berbasis Komoditas Unggulan Daerah di Kabupaten Lembata. Peraturan Bupati Lembata Nomor 8 Tahun 2021. 2021 [cited 13 July 2023]. Available from: https://peraturan.bpk.go.id/Home/Details/218333/perbup-kab-lembata-no-8-tahun-2021.

70. Tumewu R, Baruwadi MH, Halid A. Alokasi waktu kerja dan waktu luang petani jagung di Kecamatan Tilamuta Kabupaten Boalemo. Jurnal Ilmiah Agribisnis. 2021;6(1):71-7.
71. Siswanti, Wijayanti Y. Faktor risiko lingkungan kejadian kusta. Higeia. 2018;2(3). Doi: 10.15294/higeia/v2i3/23619.
72. Ratnawati R. Faktor-faktor yang berhubungan dengan risiko kejadian penyakit kusta (Morbus Hansen). 2-TRIK: Tunas-tunas Riset Kesehatan. 2016;6(3): 103-109.
73. Edi GFA, Azizah R. Analysis of environmental risk factors for leprosy in Indonesian Society: A Meta-Analysis. Poltekita: Jurnal Ilmu Kesehatan. 2023;17(1): 105-113.
74. Pollo JY, Tondobala L, Sela RLE. Ketersediaan infrastruktur permukiman kumuh pesisir studi kasus: Desa Likupang Dua dan Desa Likupang Kampung Ambong, Kecamatan Likupang Timur, Kabupaten Minahasa Utara, Provinsi Sulawesi Utara. Spasial. 2017;4(1). Doi: 10.35793/sp.v4i1.14870.
75. Pebakirang A, Tanudjaja L, Sumarauw JSF. Perencanaan sistem penyediaan air bersih di Desa Munte Kecamatan Likupang Barat Kabupaten Minahasa Utara. Jurnal Sipil Statik. 2015;3(8): 531-542.
76. Fadlila A, Nurzila U, Adriyani R. The relationship between physical conditions of house and sanitation with leprosy case in patients at Sumberglagah Mojokerto Hospital. The Indonesian Journal of Public Health. 2022;17(3): 395-405. Doi: 10.20473/ijph.vl17i3.2022.395-405.
77. Kasiadi Y, Kawatu PAT, Langi FFLG. Faktor-Faktor yang berhubungan dengan gangguan kulit pada nelayan di Desa Kalinaun Kecamatan Likupang Timur Kabupaten Minahasa Utara. Jurnal KESMAS. 2018;7(5).
78. Khanna D, de Wildt G, de Souza Duarte Filho LA, Bajaj M, Lai JF, Gardiner E, et al. Improving treatment outcomes for leprosy in Pernambuco, Brazil: a qualitative study

exploring the experiences and perceptions of retreatment patients and their carers. BMC Infectious Diseases. 2021;282. Doi: 10.1186/s12879-021-05980-5.

79. Wicaksono A, Faisya HAF, Budi IS. Hubungan lingkungan fisik rumah dan karakteristik responden dengan penyakit kusta klinis di Kota Bandar Lampung tahun 2015. Jurnal Ilmu Kesehatan Masyarakat. 2015;6(3).

Supplementary Information

S1 Data. Raw materials for baseline characteristics, bivariate, and comparison analyses. Sheet Data was used as for Table 2 (demographics of leprosy patients) and Table 3 (bivariate analysis of age and other variables). Sheet Fig3A, Fig3B, Fig3C, Fig3D, Fig3E consist of Total SALSA scores, SALSA-Work scores, JTT tests in dominant and non-dominant hand in seconds, respectively, and TUG in seconds of the population according to educational level, respectively. Sheet Fig4A, Fig4B, Fig4C, Fig4D, Fig4E, Fig4F contain Total SALSA scores, SALSA-Work scores, SALSA-Dexterity scores, P-scale, JTT in dominant and non-dominant hands in the population divided according to the WHO grading of hand disabilitiy, respectively. Sheet Fig5A, Fig5B, Fig5C contain Total SALSA scores, SALSA-Mobility scores, and P-scale in the population divided according to the WHO grading of foot disability, respectively. Fig6 contain Total SALSA scores, SALSA-mobility, -work, -dexterity scores, P-scale, JTT in both hands in seconds, and TUG in seconds of all population in the study.

Click here to access/download;Figure;Fig4F.tif

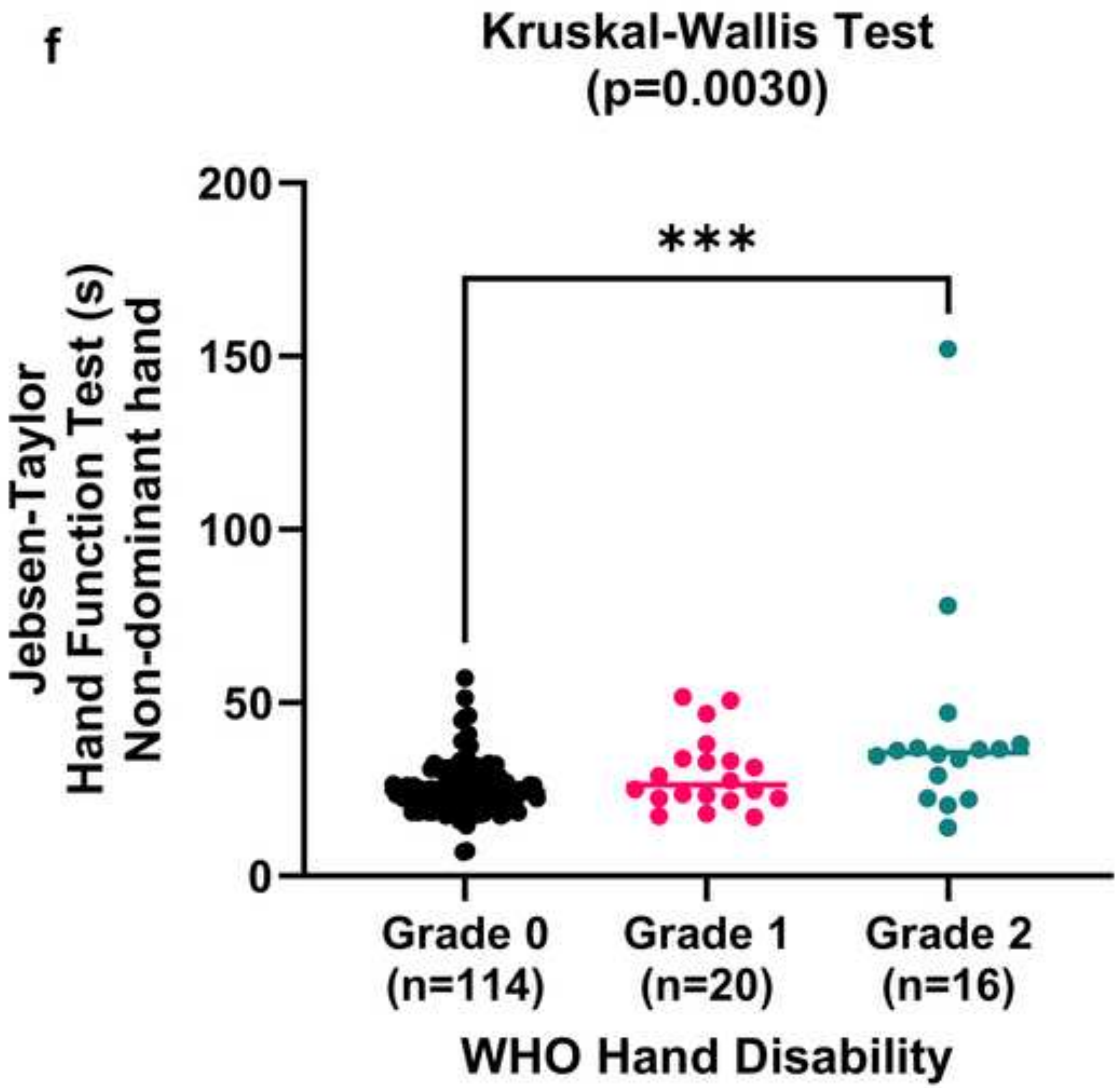

Figure 2

Click here to access/download;Figure;Fig2.tif

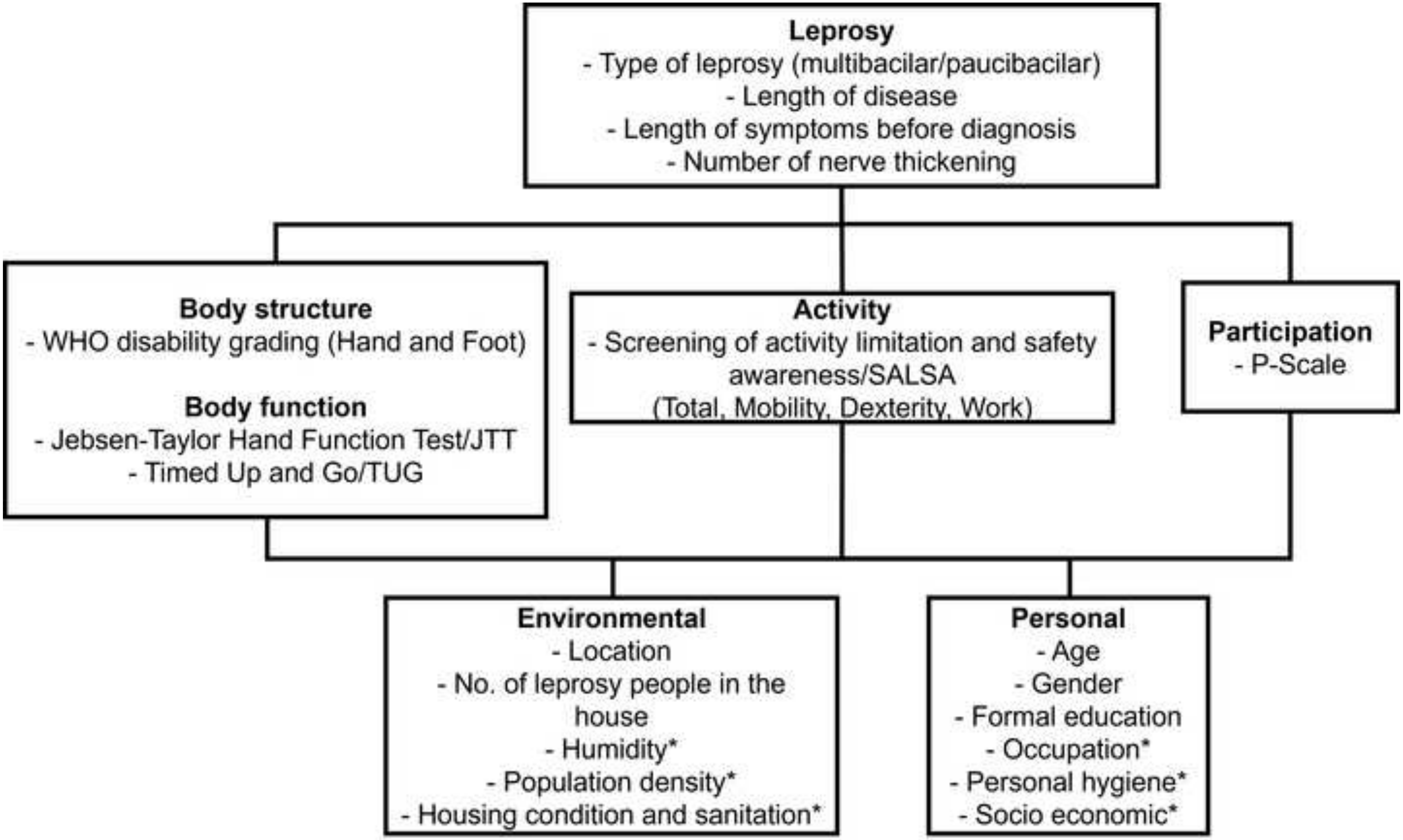

Figure 3A

Click here to access/download;Figure;Fig3A.tif

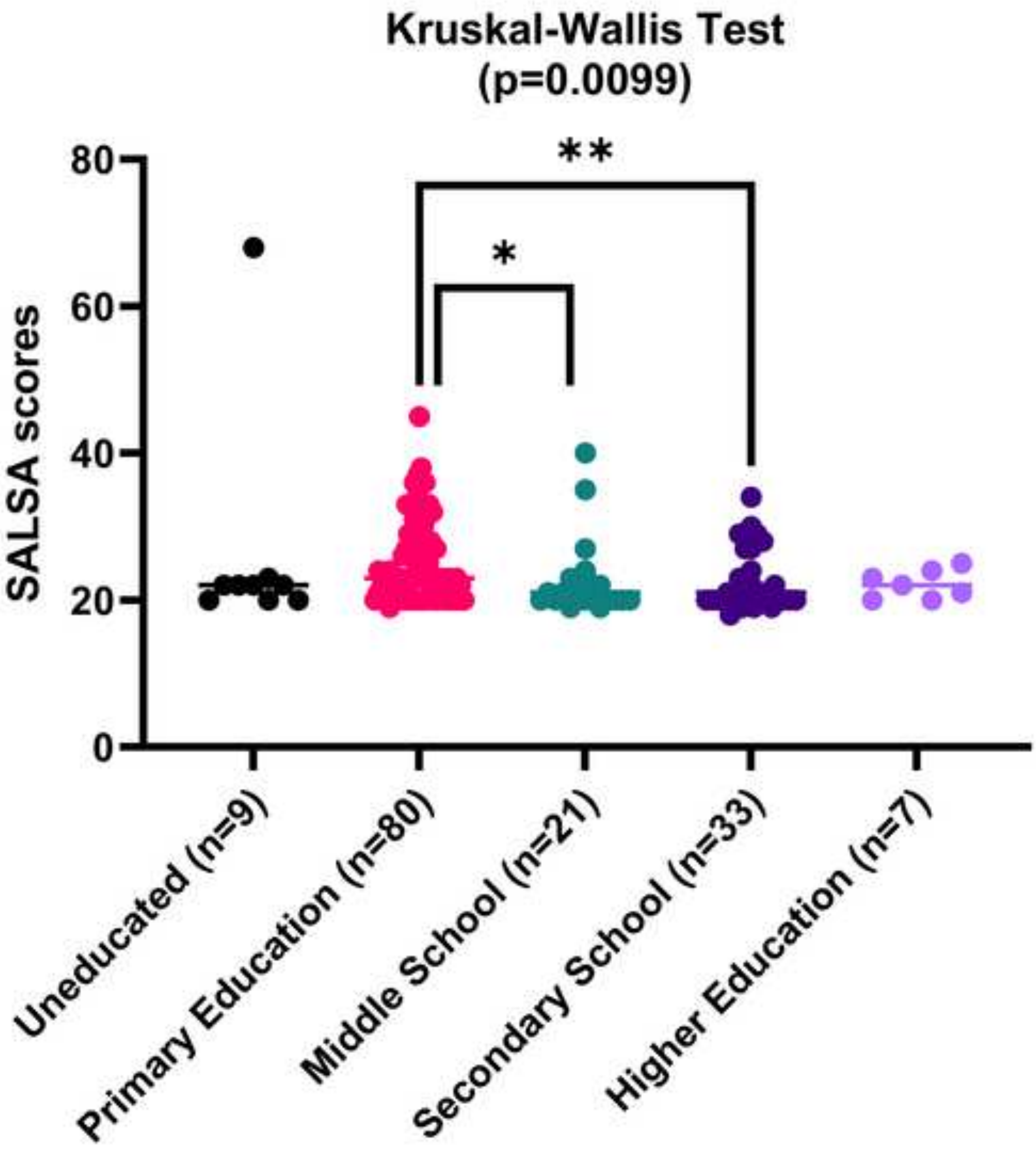

Figure 3B

Click here to access/download;Figure;Fig3B.tif

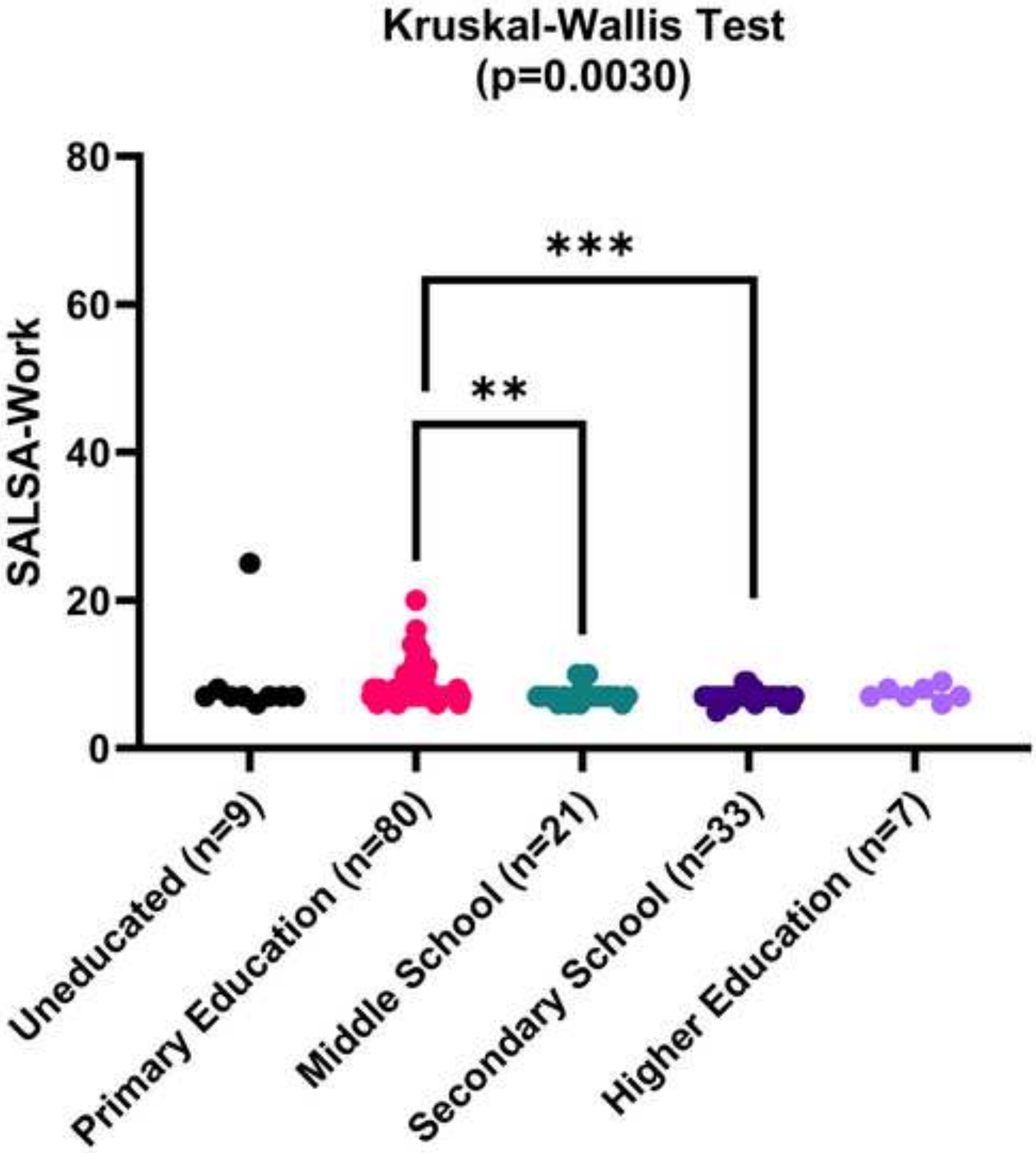

Figure 3C

Click here to access/download;Figure;Fig3C.tif

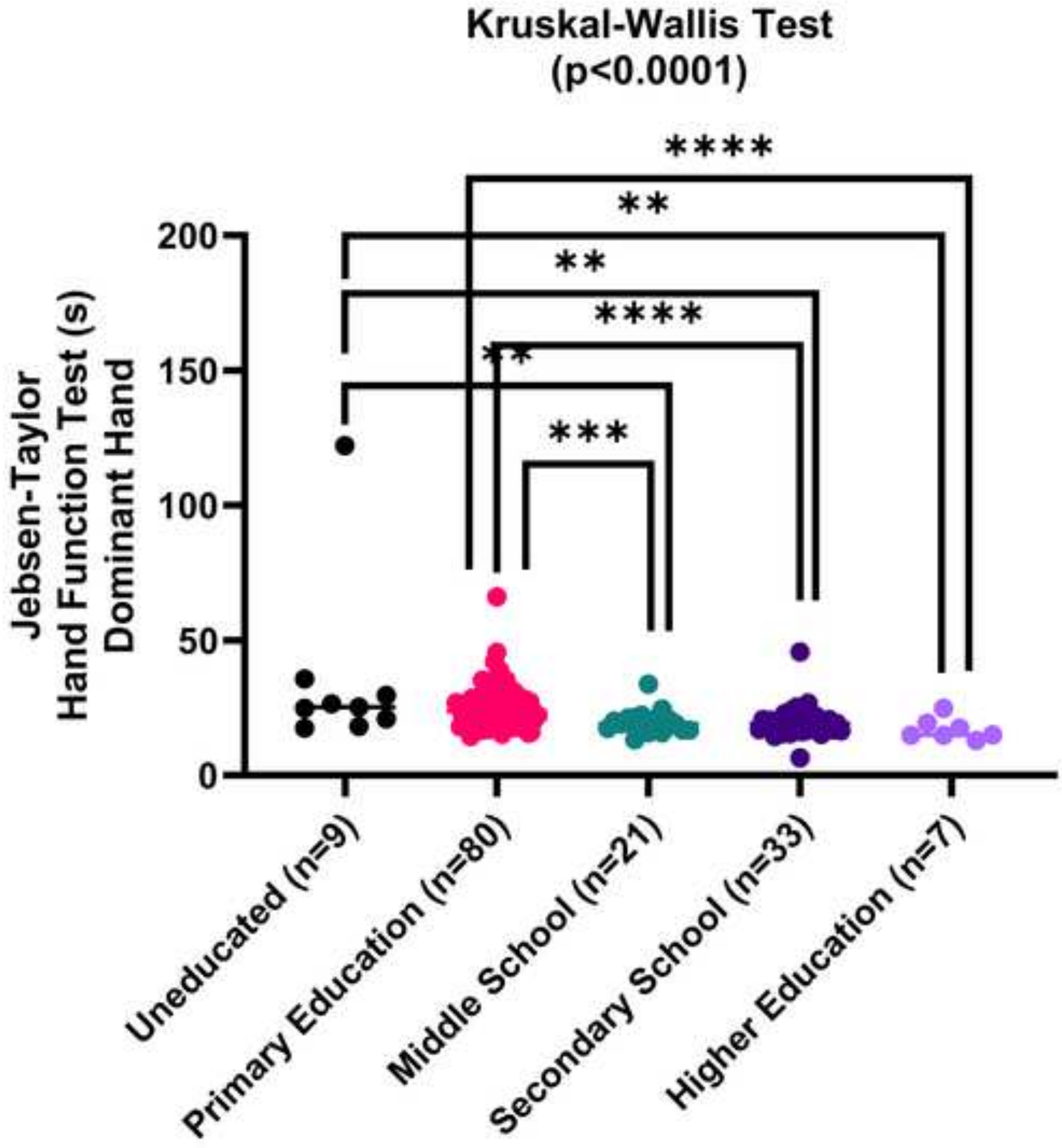

Figure 3D

Click here to access/download;Figure;Fig3D.tif

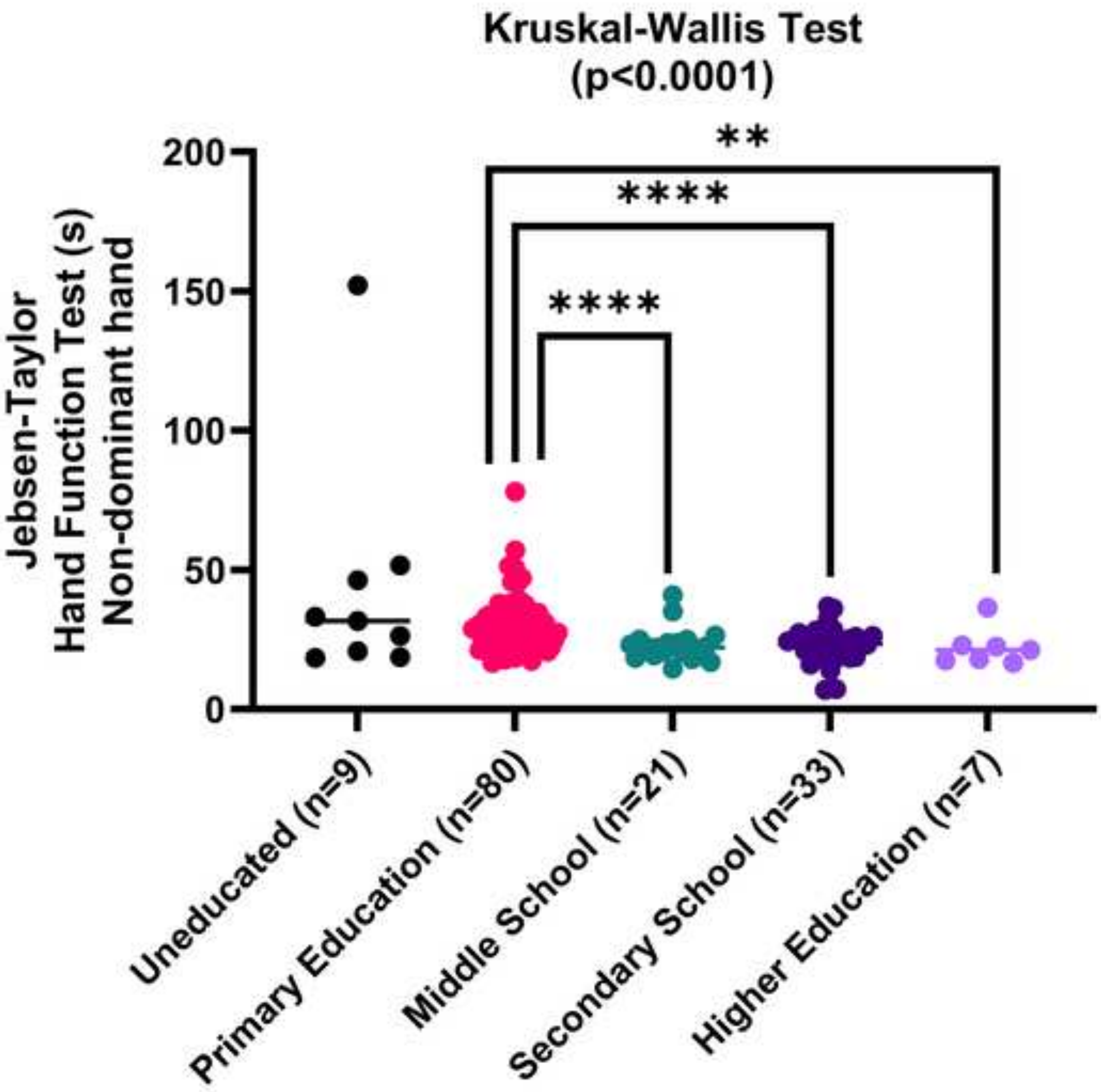

Click here to access/download;Figure;Fig3E.tif

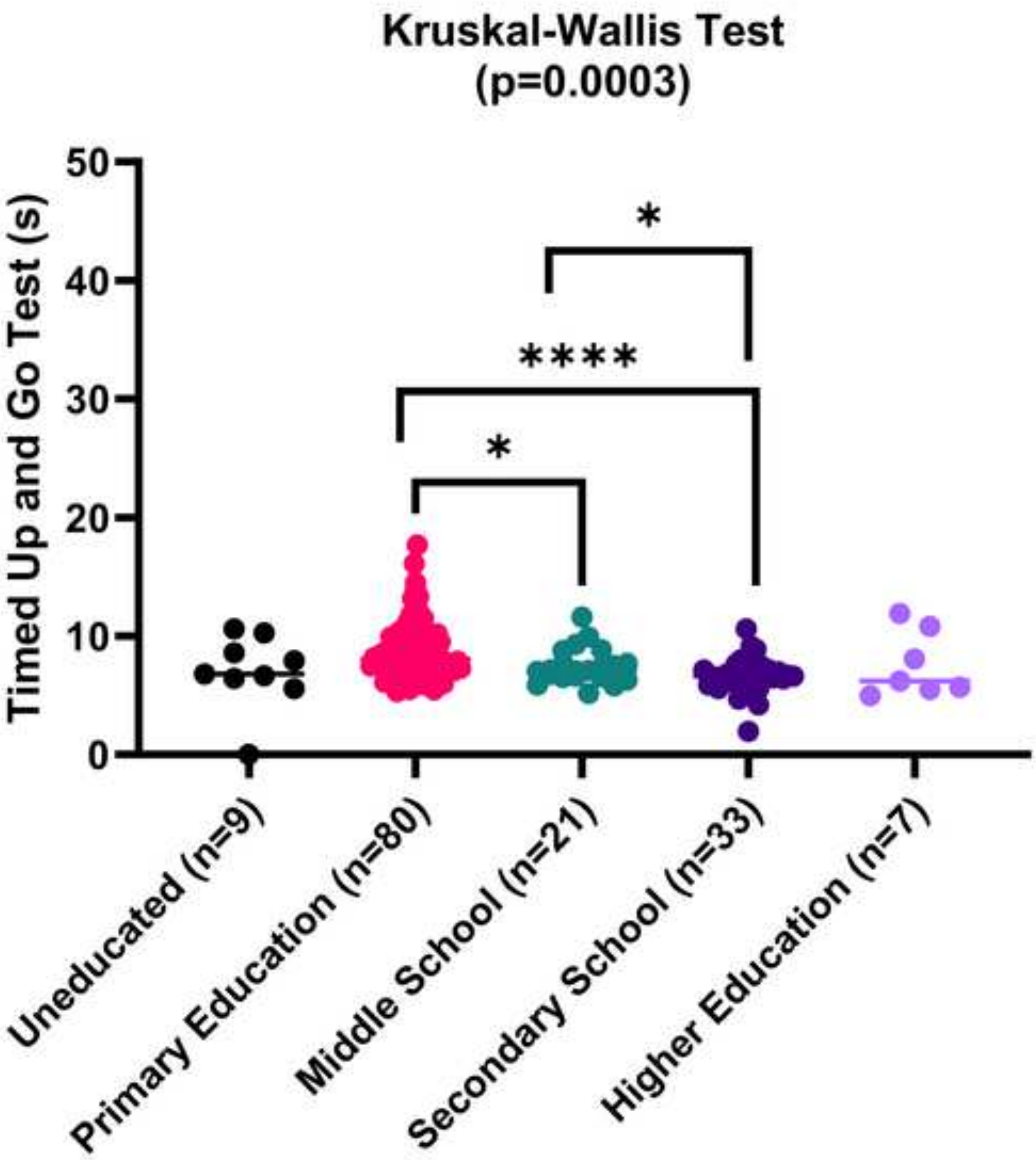

Figure 4A

Click here to access/download;Figure;Fig4A.tif

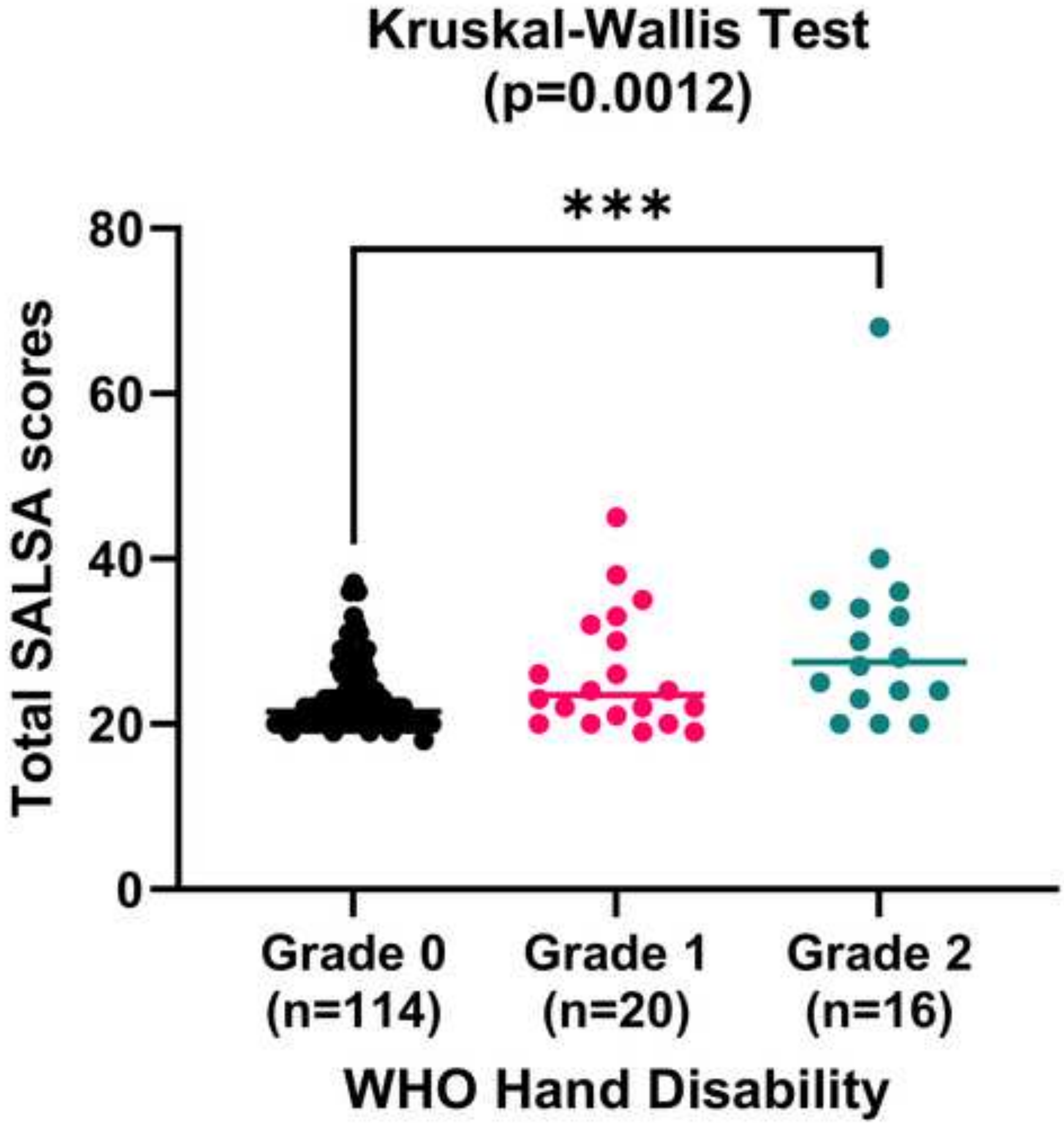

Figure 4B

Click here to access/download;Figure;Fig4B.tif

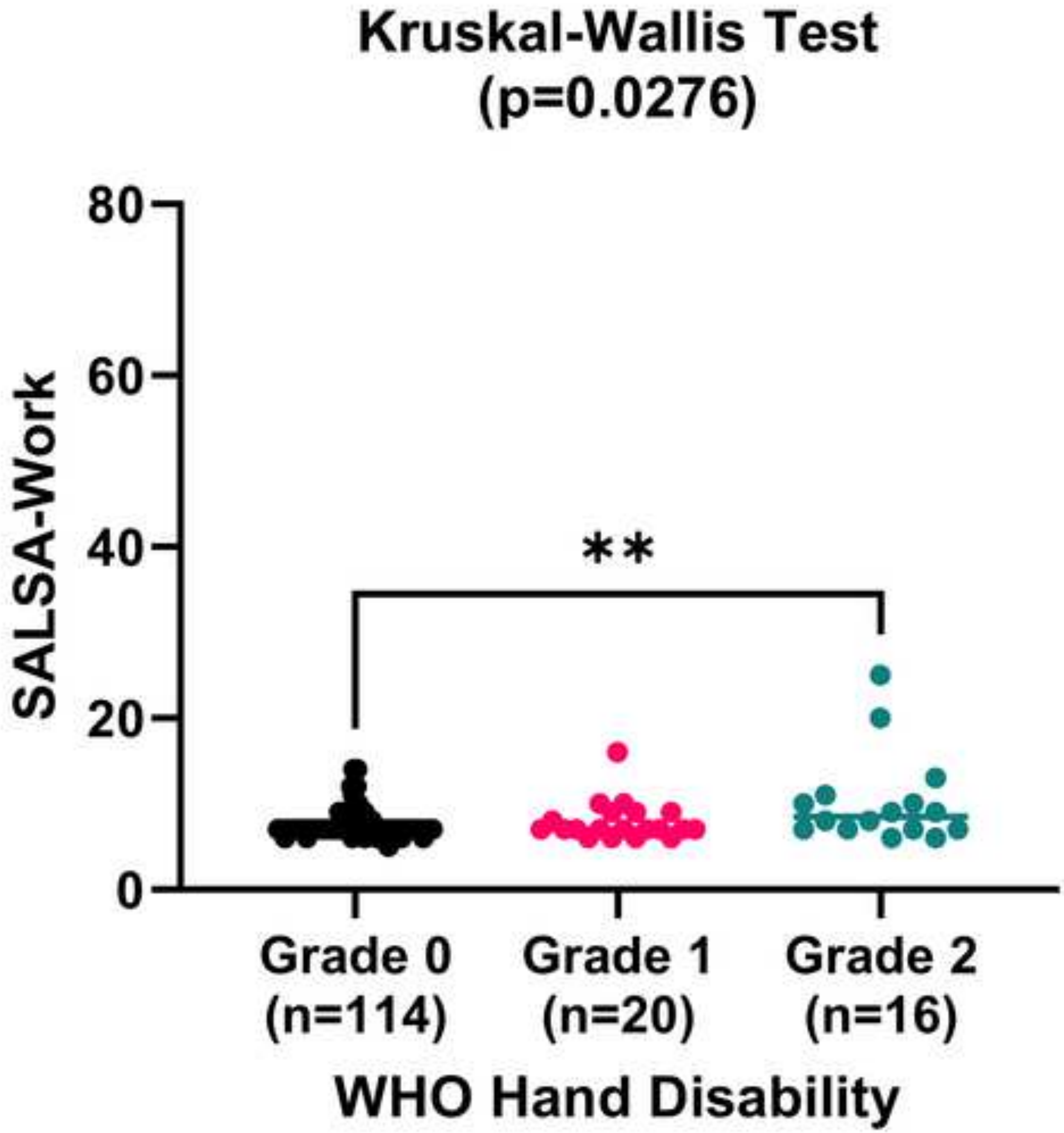

Click here to access/download;Figure;Fig4C.tif

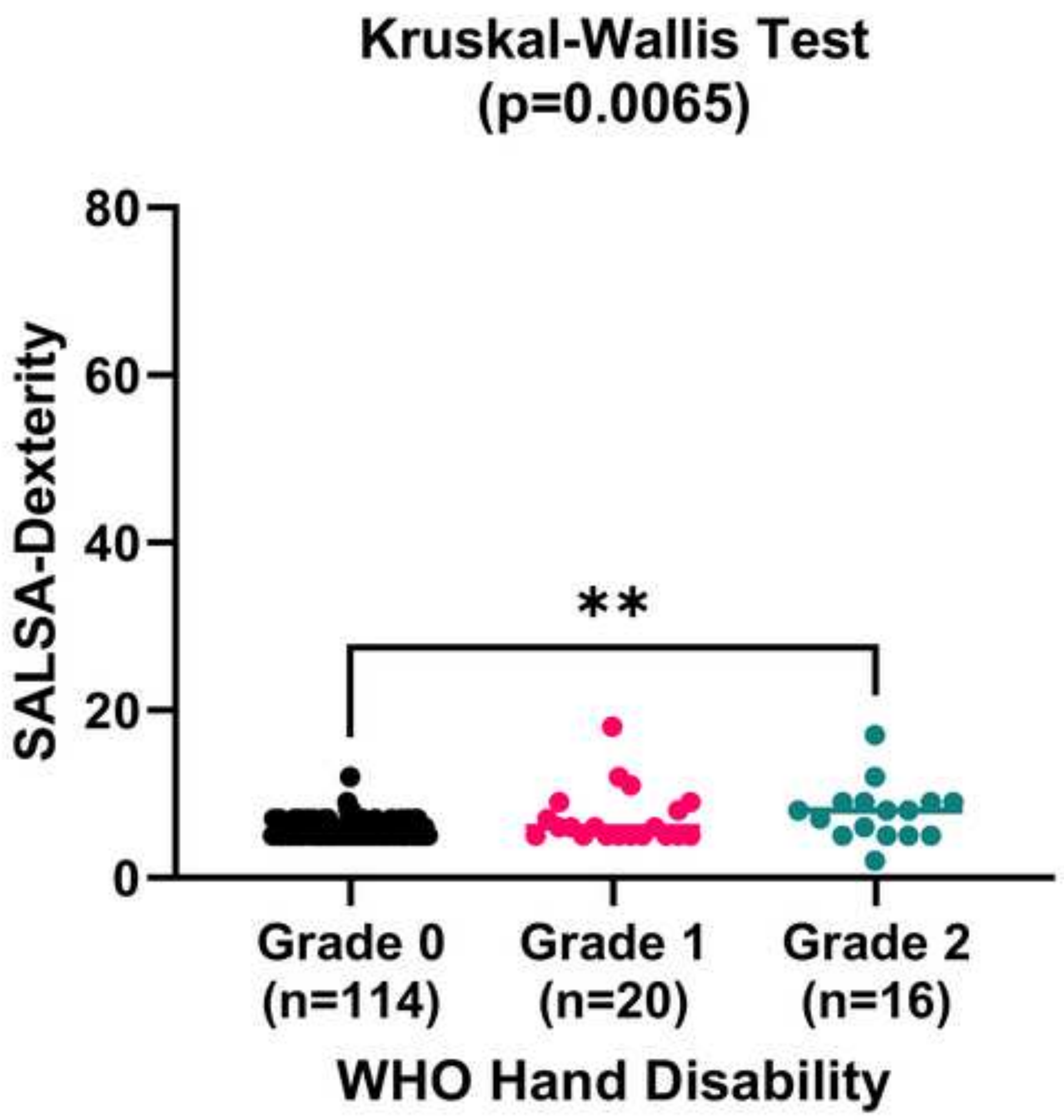

Click here to access/download;Figure;Fig4D.tif

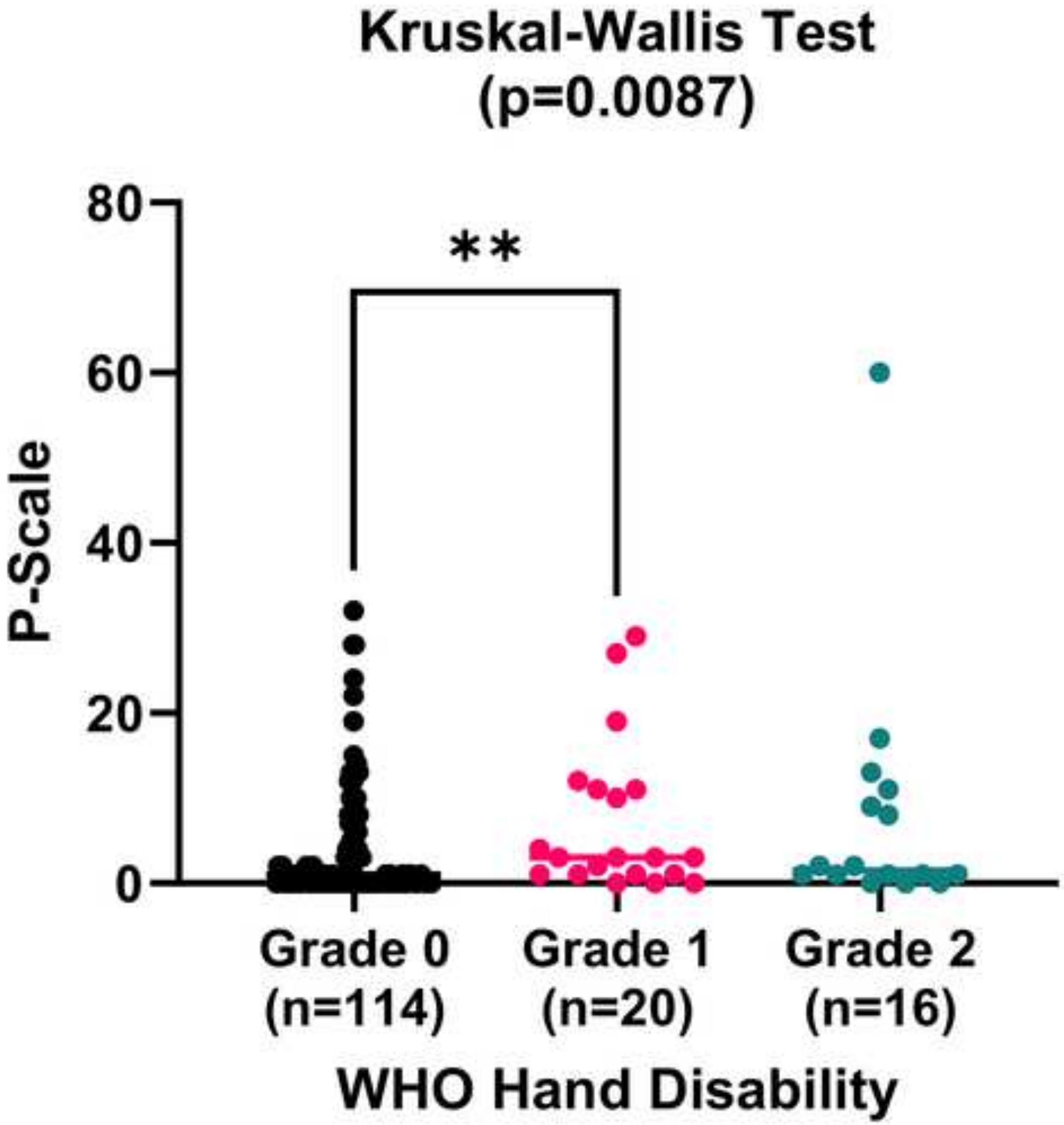

Figure 4E

Click here to access/download;Figure;Fig4E.tif

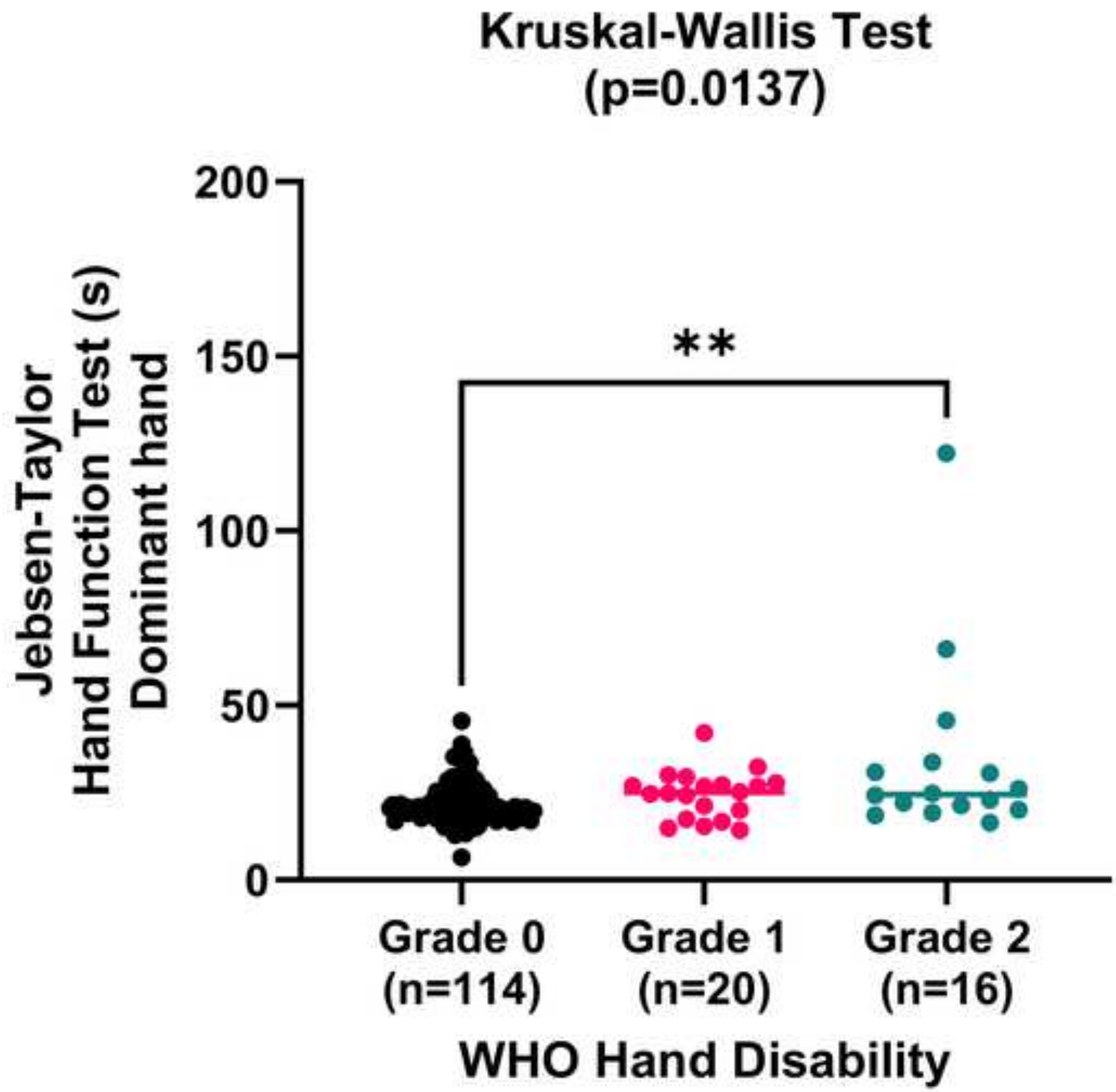

Figure 5A

Click here to access/download;Figure;Fig5A.tif

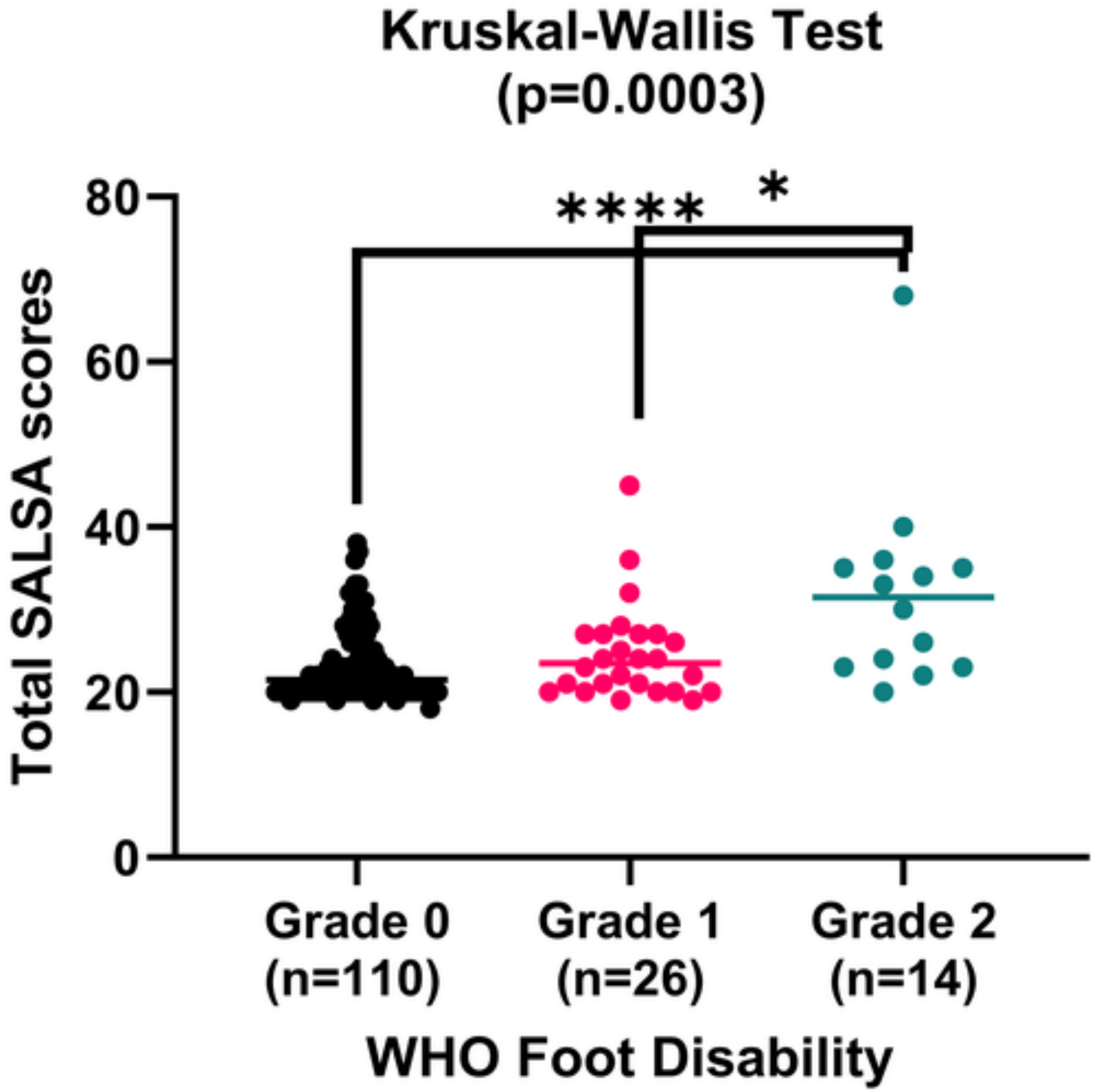

Figure 5B

Click here to access/download;Figure;Fig5B.tif

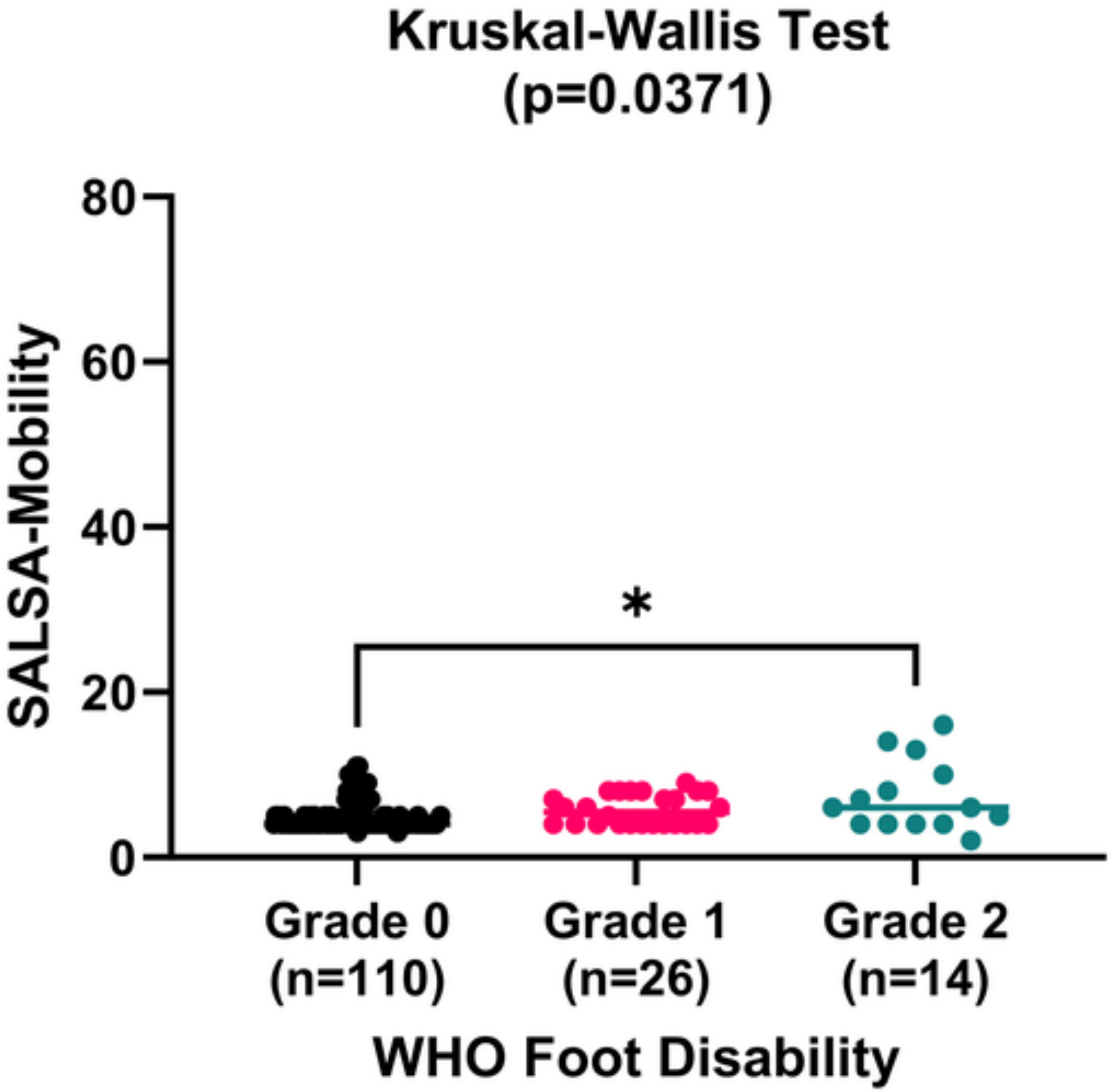

Click here to access/download;Figure;Fig5C.tif

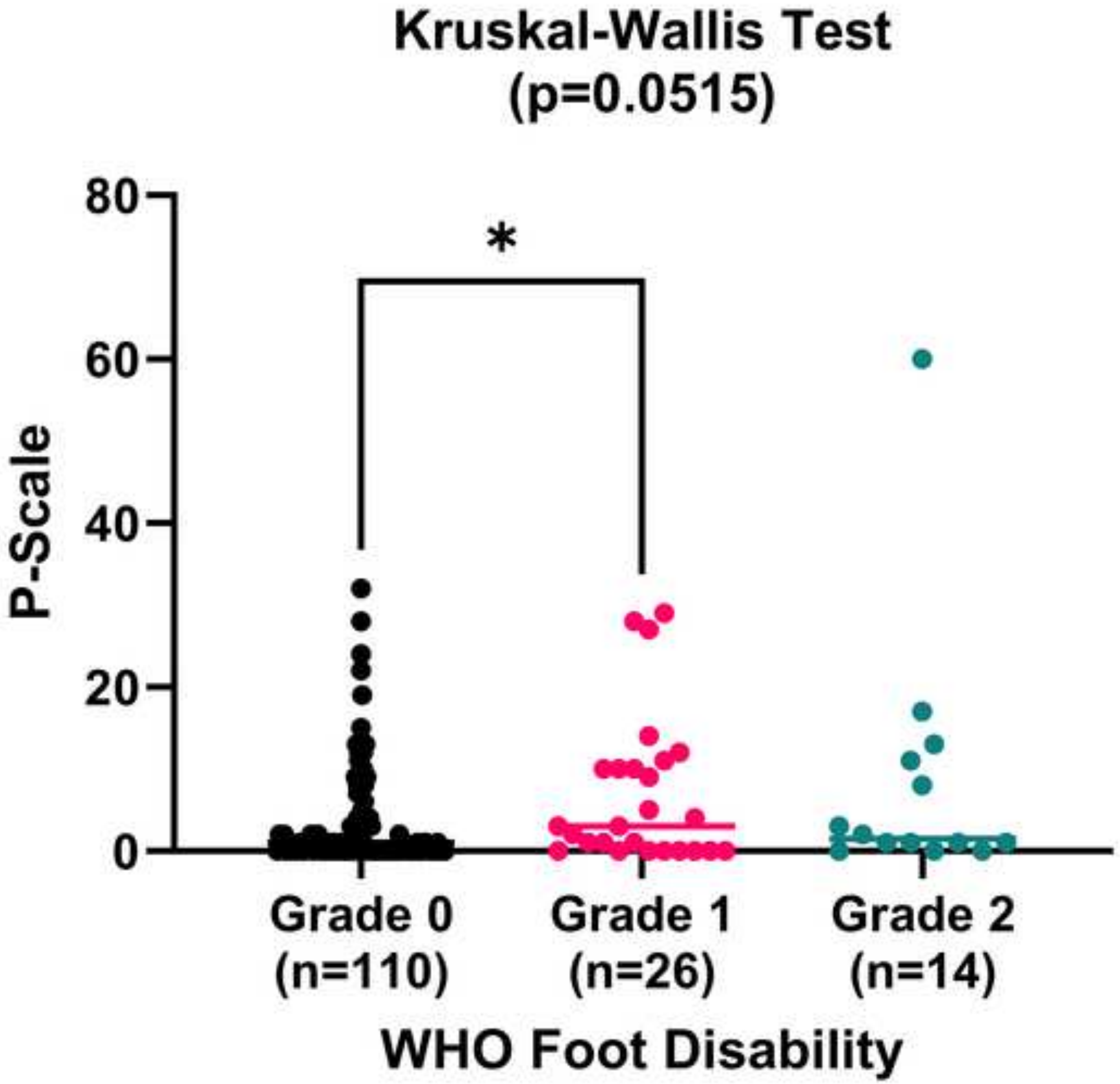

Figure 6

Click here to access/download;Figure;Fig6.tif

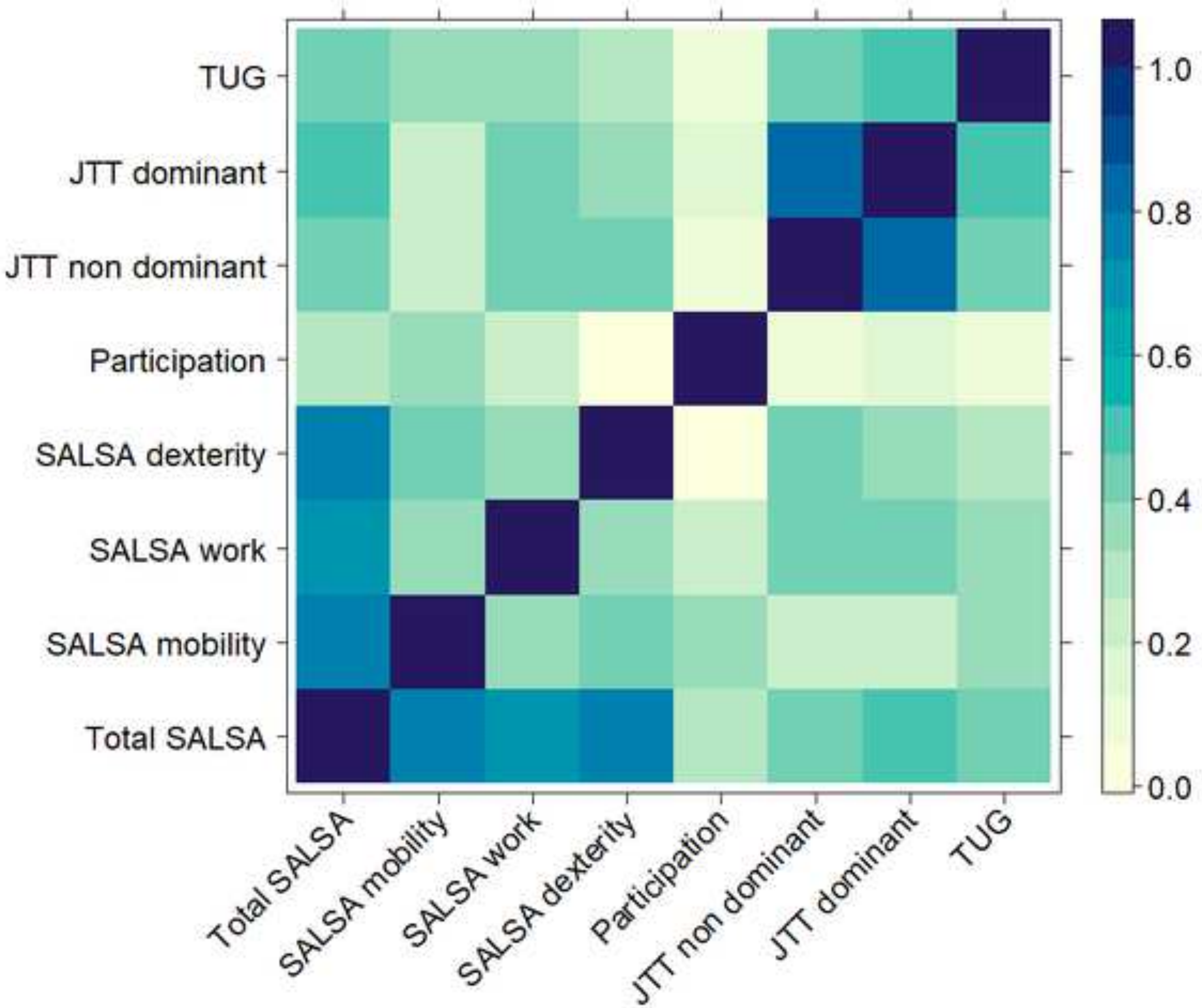

Figure 1A

Click here to access/download;Figure;Fig1A.tif

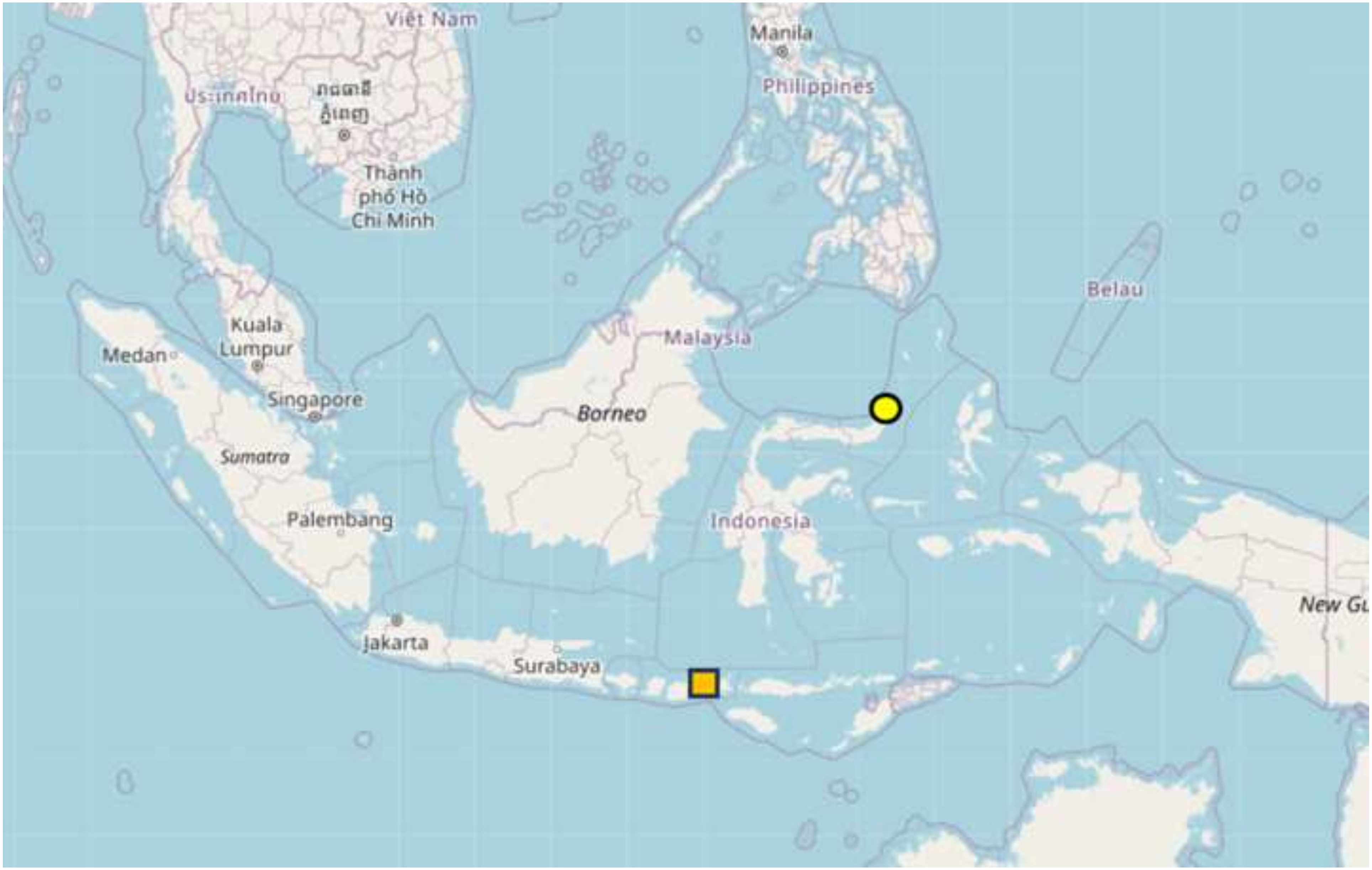

Figure 1B

Click here to access/download;Figure;Fig1B.tif

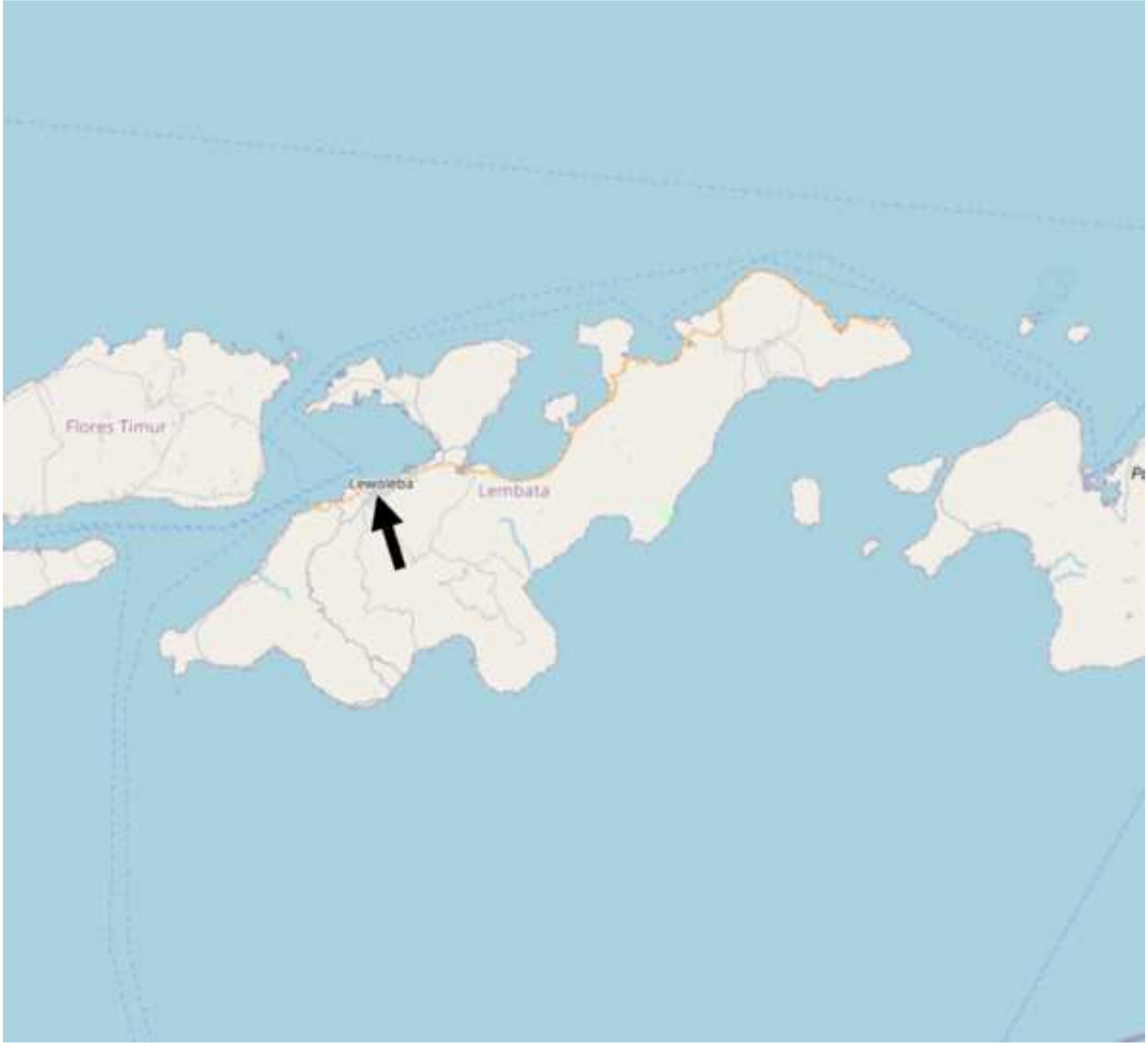

Figure 1C

Click here to access/download;Figure;Fig1C.tif

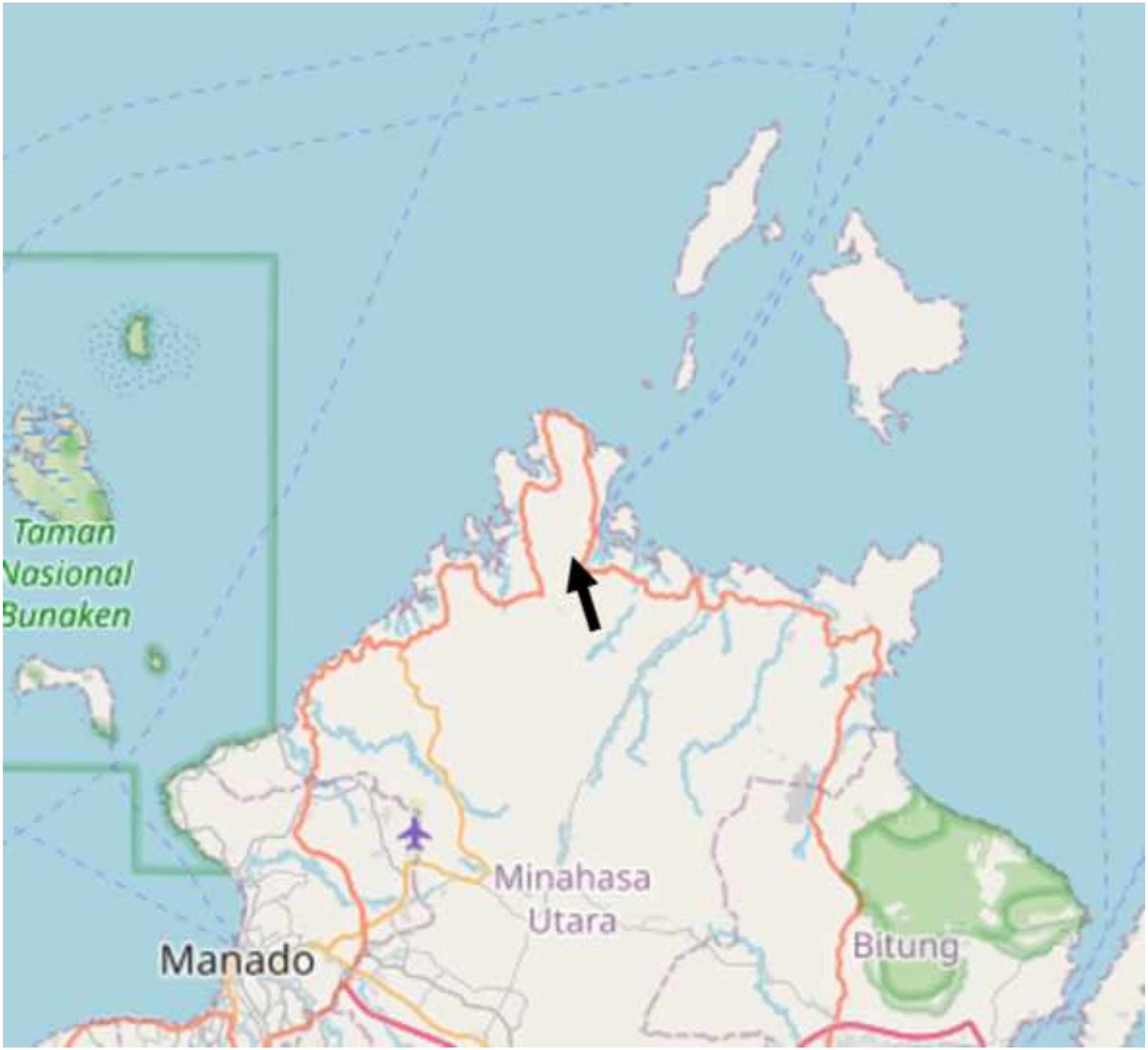

Click here to access/download
Supporting Information
Supplementary_Data 1.xlsx

Dear PLOS Neglected Tropical Disease Editor Team,

Hereby we enlist all the reviewers' comments and our responses in the table below. We have taken our time to read the comments thoroughly and many times and we have discussed with all the other authors as well. We hope that our responses can help bring the issues to light.

No	Reviewer's Comment	Response
1	In the introduction, reconcile the content of the research problems (lines 98 to 100)<br><br>"In this occasion, we want to present a study to analyze factors affecting leprosy-related disabilities involving participants from two remote areas in Indonesia: Lewoleba, Lembata, East Nusa Tenggara and Likupang, North Minahasa, North Sulawesi."<br><br>With the objectives (line 132 to 135)<br><br>"The objectives of this study are to report leprosy cases from rural and remote areas in Indonesia, map the functional disabilities of Individuals diagnosed with leprosy in Likupang and Lewoleba, Indonesia, using ICF, and to find correlation between each aspect of ICF in Indonesian population."<br><br>So that the research objective is clear and uniform.	We changed the sentences in line 98-100 and into a new research problem which similar to the objectives (line 99-103 (new manuscript)).
2	Check if there is indeed a need for maps to contextualize the study, and if necessary, revew the possibility of presenting them in the methodology.	Maps were moved to the methods.
3	Present the testable hypothesis in the study more clearly.	Hypothesis was written after the objective as:<br>"We believe that there were intercorrelation among aspects of ICF in Indonesian leprosy population"

4	In materials and methods, present the procedures adopted for the protection and secrecy of the identity of the participants.	We presented the procedures for protecting the identity of the participants by writing the subject's initials, not their name
5	In the "study and population", include the total number of patients approached, the exclusion criteria (example: neuropathies not associated with leprosy, previous disabilities, mental disabilities, incomplete data measurement for SALSA or P scale among others). In the inclusion criteria, make it clear "who the leprosy patients are, that is, time of diagnosis, if the patients are undergoing treatment or if they are undergoing post-treatment rehabilitation; there factors may interfere with the results found.	We agreed to add the inclusion and exclusion criteria from the reviewer. Those criteria were written in the methods line 182-193.
6	Describe better how the questionnaire was applied, whether it was by a previously trained professional, or the patient who filled it out, for example.	We encouraged the patients to fill in the SALSA and P-scale questionnaires independently, with the option of seeking assistance from staff members for clarifications if needed.
7	In figure 2, the variables shown in the table, "occupation, personal hygiene and socioeconomic status", were addressed in the discussion, but were not shown in the results (if this data has not been researched, remove it from the model).	We understand that information related to the participants' personal and environmental domains (e.g. occupation, personal hygiene, and socioeconomic status) were not retrieved. However, the International Classification of Functioning, Disability and Health (ICF) encouraged the
8	The method of sample size calculation and sampling were not properly explained	We used the formula for the sample size calculation using the correlation formula (already stated in the method section). In the end, the total number of samples follow those who met the criteria.
9	Why they choose two study areas? Why not more than two areas to increase the sample size (the reasons were not explained properly)	The reason why we chose Lewoleba and Likupang had been explained in the methods. We emphasized that this study is a part of a community outreach program by the KATAMATAKU team and we used the data from patients who met the inclusion criteria to be analysed in this study. However, we did

	In the study protocol, justify the reason for grouping the data from the two regions together in the results, since they are different characteristics	comparison analysis between the two areas and found that both populations were not significantly different.<br><br>The sampling methods was purposive sampling which we adjusted with the type that has been our community outreach program
10	Fisher's exact test and chi-square test are statistical methods commonly used to analyse categorical variables. It's important to note that these tests themselves do not control for confounding factors, these leading to a spurious association. To control for confounding factors in the analysis, additional methods are required for effectively controlling confounding factors and drawing valid conclusions	We have performed additional binary logistic regression analyses in statistically significant Fisher's exact tests. We also adjusted for the confounders (listed in the Statistical Analyses Method section).
11	Is there any data for length of disease? Duration of sickness? If not, should be put to limitation section.	Yes, we have included these data in our result section to be analysed with the other data. The reason why we did not include the data in the first place was because some of the data were not disclosed by the patients
12	The figures and tables are used properly but not self-explanatory (what, where, when)	We changed some points of the tables and figure as:<br>Table 1:<br>- Contains of table were pretty clear<br>- The inner rows would be removed<br>- Sub-titles were bolded<br>- Items of the tables were made using tabs<br><br>Figures:<br>- explanations of the figures were added bellow each figure<br>- Title of the figures were collated into one title<br><br>We felt that the figures are already self-explanatory, we could not put too many details (median $\pm$ range) in the figure as it will make the figure to be too crowded and difficult to understand.
13	References number were put after full stop	Yes, we had moved the references number before the full stop.

14	SALSA score validation in Indonesia? Indonesian version? Was the questionnaire self-filled or filled in by the interviewer?	The SALSA questionnaire was already available in Indonesian version. We list some publications related to the use of SALSA score in Indonesia.<br>- The SALSA Collaborative Study Group. SALSA scale (Screening Activity Limitation and Safety Awareness) users manual version 1.1 [Internet]. Netherlands; 2010 [cited 2023 September 20]. Available from: https://www.infontd.org/toolkits/nmd-toolkit/salsa-scale.<br>- Menaldi SL, Harini M, Nelfidayani N, Irawati Y, Setiono S, Wahyuni LK, Rahayu T, Andayani G, Friska D, Nugraha B. Functional activity limitation of leprosy cases in an endemic area in Indonesia and recommendations for integrated participation program in society. PLoS Negl Trop Dis. 2022 Aug 19;16(8):e0010646. doi: 10.1371/journal.pntd.0010646.<br>- Akkerman L. The quantitative cultural validation of three tools among people affected by Leprosy in West Java, Indonesia. VU University Amsterdam. 2016:53. Available from: https://www.infontd.org/resource/quantitative-cultural-validation-three-tools-among-people-affected-leprosy-west-java.
15	P-scale validation in Indonesia? Indonesian version? Was the questionnaire self-filled or filled in by the interviewer?	Yes, P-scale is available in Indonesian version. Here we list some publications related to utilizations of p-scale in Indonesian population.<br>- The Participation Scale Development Team. Participation scale users manual version 6.0 [Internet]. Netherlands; 2010 [cited 2023 September 20]. Available from: https://www.infontd.org/toolkits/ntd-morbidity-and-disability-toolkit/participation-scale-p-scale.<br>- Kelders R, van Brakel W, Beise K, Irwanto. Testing and validating a simplified scale to measure social participation of people with disabilities in Indonesia. Disabil Rehabil. 2012;34(8):638-46. doi: 10.3109/09638288.2011.615369.<br>- Akkerman L. The quantitative cultural validation of three tools among people affected by Leprosy in West Java, Indonesia. VU University Amsterdam. 2016:53. Available from: https://www.infontd.org/resource/quantitative-

		cultural-validation-three-tools-among-people-affected-leprosy-west-java
16	All the tables should be analyzed further using Mann-Whitney U to know about the correlation	Yes, all the comparison analysis in the resubmitted manuscript has been re-analyzed using Mann-Whitney U.
17	Figure 5 should be figure 4	We had changed the numeration of all figures and adjust them systematically.
18	Explain more about line 272-274	We change the sentence and summarize the results: "In this study, we found that hand and foot disability affected the activity limitation and participation restriction domains in this population. Hand disability also affected body function domains. Both participation restriction and activity limitation had significant correlation to one another. However, activity limitations also had significant correlation with upper and lower body functions. Activity limitation and body functions were affected by age and educational levels. We also found that locations of the population and the types of leprosy did not significantly affect disability progression. All in all, our results imply that the body structure and body function domain in ICF affected activity limitation and participation restriction and vice versa in leprosy patients."
19	Line 302-305<br>Our bivariate analysis found that the number of nerve enlargement in leprosy patients has weak but positive correlations to functional activity.<br>304 limitation and social participation restriction, which was represented by SALSA scale (r=0,165, 305 p=0,044) and P-scale (r=0,167, p=0,041), respectively.<br><br>Are they not supposed to be correlated according to Hinkle et al (2003)?	We include the reference in the method (statistical analysis section) and change the sentence into:<br><br>"However, our bivariate analyses showed that the number of nerve enlargement in leprosy did not correlate to activity limitation and participation restriction which was represented by SALSA scale (r=0,165, p=0,044) and P-Scale (r=0,167, p=0,041), respectively."
20	Response for the comment of line 323 that whether the high participation in the society may be due to the population being in the nested/exclusive groups.	We don't have sufficient data to confirm that those participants came from nested or exclusive groups.

We have answered all the feedbacks from the reviewers. This manuscript is not under consideration elsewhere. All authors have contributed significantly to the manuscript, have agreed to its submission to PLOS Neglected Tropical Disease in its present form and will observe the terms of the License to Publish, if the manuscript is accepted. There are no conflicts of interest. We hope that the manuscript will be put into consideration for publication in PLOS Neglected Tropical Disease. Thank you for your consideration.

Sincerely,

Dr. dr. Luh Karunia Wahyuni, Sp.KFR(K)

Paediatric Division, Dept. of Physical Medicine and Rehabilitation

Faculty of Medicine Universitas Indonesia

Click here to access/download
Revised Article with Changes Highlighted
Manuscript (marked)(1).docx
