# Supplementary material for: The International Classification of Functioning, Disability and Health to map leprosy-related disability in rural and remote areas in Indonesia
Source: PLoS Negl Trop Dis. 2024 May 21;18(5):e0011539. doi: 10.1371/journal.pntd.0011539 (PMC11161106; doi:10.1371/journal.pntd.0011539)
Supplement: Attachment — Submitted filename: Response to reviewer PLOS Neg Trop Dis(1).docx [file pntd.0011539.s019.docx]

Dear PLOS Neglected Tropical Disease Editor Team,

Hereby we enlist all the reviewers’ comments and our responses in the table below. We have taken our time to read the comments thoroughly and many times and we have discussed with all the other authors as well. We hope that our responses can help bring the issues to light.

| No | Reviewer’s Comment | Response |
| --- | --- | --- |
| 1 | In the introduction, reconcile the content of the research problems (lines 98 to 100)  “In this occasion, we want to present a study to analyze factors affecting leprosy-related disabilities involving participants from two remote areas in Indonesia: Lewoleba, Lembata, East Nusa Tenggara and Likupang, North Minahasa, North Sulawesi.”  With the objectives (line 132 to 135)  “The objectives of this study are to report leprosy cases from rural and remote areas in Indonesia, map the functional disabilities of Individuals diagnosed with leprosy in Likupang and Lewoleba, Indonesia, using ICF, and to find correlation between each aspect of ICF in Indonesian population.”  So that the research objective is clear and uniform. | We changed the sentences in line 98-100 and into a new research problem which similar to the objectives (line 99-103 (new manuscript)). |
| 2 | Check if there is indeed a need for maps to contextualize the study, and if necessary, revew the possibility of presenting them in the methodology. | Maps were moved to the methods. |
| 3 | Present the testable hypothesis in the study more clearly. | Hypothesis was written after the objective as:  “We believe that there were intercorrelation among aspects of ICF in Indonesian leprosy population” |
| 4 | In materials and methods, present the procedures adopted for the protection and secrecy of the identity of the participants. | We presented the procedures for protecting the identity of the participants by writing the subject’s initials, not their name |
| 5 | In the “study and population”, include the total number of patients approached, the exclusion criteria (example: neuropathies not associated with leprosy, previous disabilities, mental disabilities, incomplete data measurement for SALSA or P scale among others). In the inclusion criteria, make it clear “who the leprosy patients are, that is, time of diagnosis, if the patients are undergoing treatment or if they are undergoing post-treatment rehabilitation; there factors may interfere with the results found. | We agreed to add the inclusion and exclusion criteria from the reviewer. Those criteria were written in the methods line 182-193. |
| 6 | Describe better how the questionnaire was applied, whether it was by a previously trained professional, or the patient who filled it out, for example. | We encouraged the patients to fill in the SALSA and P-scale questionnaires independently, with the option of seeking assistance from staff members for clarifications if needed. |
| 7 | In figure 2, the variables shown in the table,  “occupation, personal hygiene and socioeconomic status”, were addressed in the discussion, but were not shown in the results (if this data has not been researched, remove it from the model). | We understand that information related to the participants’ personal and environmental domains (e.g. occupation, personal hygiene, and socioeconomic status) were not retrieved. However, the International Classification of Functioning, Disability and Health (ICF) encouraged the |
| 8 | The method of sample size calculation and sampling were not properly explained | We used the formula for the sample size calculation using the correlation formula (already stated in the method section). In the end, the total number of samples follow those who met the criteria. |
| 9 | Why they choose two study areas? Why not more than two areas to increase the sample size (the reasons were not explained properly)  In the study protocol, justify the reason for grouping the data from the two regions together in the results, since they are different characteristics | The reason why we chose Lewoleba and Likupang had been explained in the methods. We emphasized that this study is a part of a community outreach program by the KATAMATAKU team and we used the data from patients who met the inclusion criteria to be analysed in this study. However, we did comparison analysis between the two areas and found that both populations were not significantly different.  The sampling methods was purposive sampling which we adjusted with the type that has been our community outreach program |
| 10 | Fisher's exact test and chi-square test are statistical methods commonly used to analyse categorical variables. It's important to note that these tests themselves do not control for confounding factors, these leading to a spurious association. To control for confounding factors in the analysis, additional methods are required for effectively controlling confounding factors and drawing valid conclusions | We have performed additional binary logistic regression analyses in statistically significant Fisher’s exact tests. We also adjusted for the confounders (listed in the Statistical Analyses Method section). |
| 11 | Is there any data for length of disease? Duration of sickness? If not, should be put to limitation section. | Yes, we have included these data in our result section to be analysed with the other data. The reason why we did not include the data in the first place was because some of the data were not disclosed by the patients |
| 12 | The figures and tables are used properly but not self-explanatory (what, where, when) | We changed some points of the tables and figure as:  Table 1:  - Contains of table were pretty clear  - The inner rows would be removed  - Sub-titles were bolded  - Items of the tables were made using tabs  Figures:  - explanations of the figures were added bellow each figure  - Title of the figures were collated into one title  We felt that the figures are already self-explanatory, we could not put too many details (median ± range) in the figure as it will make the figure to be too crowded and difficult to understand. |
| 13 | References number were put after full stop | Yes, we had moved the references number before the full stop. |
| 14 | SALSA score validation in Indonesia? Indonesian version? Was the questionnaire self-filled or filled in by the interviewer? | The SALSA questionnaire was already available in Indonesian version. We list some publications related to the use of SALSA score in Indonesia.   - The SALSA Collaborative Study Group. SALSA scale (Screening Activity Limitation and Safety Awareness) users manual version 1.1 [Internet]. Netherlands; 2010 [cited 2023 September 20]. Available from: https://www.infontd.org/toolkits/nmd-toolkit/salsa-scale. - Menaldi SL, Harini M, Nelfidayani N, Irawati Y, Setiono S, Wahyuni LK, Rahayu T, Andayani G, Friska D, Nugraha B. Functional activity limitation of leprosy cases in an endemic area in Indonesia and recommendations for integrated participation program in society. PLoS Negl Trop Dis. 2022 Aug 19;16(8):e0010646. doi: 10.1371/journal.pntd.0010646. - Akkerman L. The quantitative cultural validation of three tools among people affected by Leprosy in West Java, Indonesia. VU University Amsterdam. 2016:53. Available from: https://www.infontd.org/resource/quantitative-cultural-validation-three-tools-among-people-affected-leprosy-west-java. |
| 15 | P-scale validation in Indonesia? Indonesian version? Was the questionnaire self-filled or filled in by the interviewer? | Yes, P-scale is available in Indonesian version. Here we list some publications related to utilizations of p-scale in Indonesian population.   - The Participation Scale Development Team. Participation scale users manual version 6.0 [Internet]. Netherlands; 2010 [cited 2023 September 20]. Available from: https://www.infontd.org/toolkits/ntd-morbidity-and-disability-toolkit/participation-scale-p-scale. - Kelders R, van Brakel W, Beise K, Irwanto. Testing and validating a simplified scale to measure social participation of people with disabilities in Indonesia. Disabil Rehabil. 2012;34(8):638-46. doi: 10.3109/09638288.2011.615369. - Akkerman L. The quantitative cultural validation of three tools among people affected by Leprosy in West Java, Indonesia. VU University Amsterdam. 2016:53. Available from: https://www.infontd.org/resource/quantitative-cultural-validation-three-tools-among-people-affected-leprosy-west-java |
| 16 | All the tables should be analyzed further using Mann-Whitney U to know about the correlation | Yes, all the comparison analysis in the resubmitted manuscript has been re-analyzed using Mann-Whitney U. |
| 17 | Figure 5 should be figure 4 | We had changed the numeration of all figures and adjust them systematically. |
| 18 | Explain more about line 272-274 | We change the sentence and summarize the results:  “In this study, we found that hand and foot disability affected the activity limitation and participation restriction domains in this population. Hand disability also affected body function domains. Both participation restriction and activity limitation had significant correlation to one another. However, activity limitations also had significant correlation with upper and lower body functions. Activity limitation and body functions were affected by age and educational levels. We also found that locations of the population and the types of leprosy did not significantly affect disability progression. All in all, our results imply that the body structure and body function domain in ICF affected activity limitation and participation restriction and vice versa in leprosy patients.” |
| 19 | Line 302-305  Our bivariate analysis found that the number of nerve enlargement in leprosy patients has weak but positive correlations to functional activity.  304 limitation and social participation restriction, which was represented by SALSA scale (r=0,165, 305 p=0,044) and P-scale (r=0,167, p=0,041), respectively.  Are they not supposed to be correlated according to Hinkle et al (2003)? | We include the reference in the method (statistical analysis section) and change the sentence into:  “However, our bivariate analyses showed that the number of nerve enlargement in leprosy did not correlate to activity limitation and participation restriction which was represented by SALSA scale (r=0,165, p=0,044) and P-Scale (r=0,167, p=0,041), respectively.” |
| 20 | Response for the comment of line 323 that whether the high participation in the society may be due to the population being in the nested/exclusive groups. | We don’t have sufficient data to confirm that those participants came from nested or exclusive groups. |

We have answered all the feedbacks from the reviewers. This manuscript is not under consideration elsewhere. All authors have contributed significantly to the manuscript, have agreed to its submission to PLOS Neglected Tropical Disease in its present form and will observe the terms of the License to Publish, if the manuscript is accepted. There are no conflicts of interest. We hope that the manuscript will be put into consideration for publication in PLOS Neglected Tropical Disease. Thank you for your consideration.

Sincerely,

Dr. dr. Luh Karunia Wahyuni, Sp.KFR(K)

Paediatric Division, Dept. of Physical Medicine and Rehabilitation

Faculty of Medicine Universitas Indonesia
