# Supplementary material for: The International Classification of Functioning, Disability and Health to map leprosy-related disability in rural and remote areas in Indonesia
Source: PLoS Negl Trop Dis. 2024 May 21;18(5):e0011539. doi: 10.1371/journal.pntd.0011539 (PMC11161106; doi:10.1371/journal.pntd.0011539)
Supplement: Attachment — Submitted filename: Response to Reviewer PLOS NTD .docx [file pntd.0011539.s021.docx]

Dear PLOS Neglected Tropical Disease Editor Team,

Hereby we enlist all the reviewers’ comments and our responses in the table below. We have taken our time to read the comments thoroughly and many times and we have discussed with all the other authors as well. We hope that our responses can help bring the issues to light.

| No | Reviewer’s Comment | Response |
| --- | --- | --- |
| 1 | Some results shown in the figure are suggested to make it clearer, such as which one is statistically significant, you can put the symbol in the figure, not only by words and state what the symbol * means (Figure 3,4 and 5) | Explanation of symbol * was added  (line 296, 350, 366) |
| 2 | For figure 6, I suggest please to put statement which variables show the strongest correlation (r) and also the range of coefficient correlation | The strongest correlation and range of coefficient correlation were added in lines 396-412 |
| 3 | Yes, the conclusion supported with the data, limitation of the study are stated, even though not for limitation of analysis. Public Health relevance is addressed, but as suggestion, it would be better if the author mention what kind of educational and disability prevention | We add educational and disability prevention in conclusion as reviewer suggested |
| 4 | The conclusions drawn by the authors are well-supported by the data. However, it would be valuable to include deeper clinical implications and recommendations for healthcare professionals and policy developers | Recommendations and clinical implications were added in conclusion |
| 5 | The study design was well-executed and the methods were clearly described. However, the sample size could have been larger to increase generalizability | We will recommend for future study to cover bigger sample size |
| 6 | The statistical analysis appears to be sound. However, it would be helpful to include confidence intervals for the reported results. | We agree with reviewer suggestion, so we add several confidence intervals (CI) to our study results |
| 7 | It would be beneficial to provide more detailed information about participant demographics | Information about participant demographics provided were already plenty, but we will encouraged for future study to include more detailed information |
| 8 | The authors included more recent literature to strengthen the background and relevance of the study. | We try to emphasize the use of the latest literature in our study |
| 9 | The discussion section provides a comprehensive analysis and interpretation of the findings. However, it would be helpful to discuss potential implications for future research or clinical practice. | We included potential implications for future research and clinical practice in ***Conclusion*** segment |
| 10 | The paper is well-written and organized. However, some sections could be better clarified, especially in terms of terminology | Terminology used were customized, so reader could understand it better |
| 11 | The study results are significant and contribute to the existing literature. However, it would be valuable to include a discussion on the potential mechanisms underlying the observed associations | We provide potential mechanisms in ***Discussion*** segment, such as age, education background, environmental condition, personal hygiene, etc. |
| 12 | The conclusions drawn by the authors are well-supported by the data. However, it would be valuable to include deeper clinical implications and recommendations for healthcare professionals and policy developers | Recommendations and clinical implications were added in conclusion |

We have answered all the feedback from the reviewers. This manuscript is not under consideration elsewhere. All authors have contributed significantly to the manuscript, have agreed to its submission to PLOS Neglected Tropical Disease in its present form, and will observe the terms of the License to Publish if the manuscript is accepted. There are no conflicts of interest. We hope that the manuscript will be put into consideration for publication in PLOS Neglected Tropical Disease. Thank you for your consideration.

Sincerely,

Dr. dr. Luh Karunia Wahyuni, Sp.KFR(K)

Paediatric Division, Dept. of Physical Medicine and Rehabilitation

Faculty of Medicine Universitas Indonesia
