# Supplementary material for: The International Classification of Functioning, Disability and Health to map leprosy-related disability in rural and remote areas in Indonesia
Source: PLoS Negl Trop Dis. 2024 May 21;18(5):e0011539. doi: 10.1371/journal.pntd.0011539 (PMC11161106; doi:10.1371/journal.pntd.0011539)
Supplement: Attachment — Submitted filename: Response to Reviewer PLOS NTD.docx [file pntd.0011539.s022.docx]

Dear PLOS Neglected Tropical Disease Editor Team,

Hereby we enlist all the reviewers’ comments and our responses in the table below. We have taken our time to read the comments thoroughly and many times and we have discussed with all the other authors as well. We hope that our responses can help bring the issues to light.

| No | Reviewer’s Comment | Response |
| --- | --- | --- |
| 1 | The results presented clearly and concisely but the prevalence better put in 95% CI (, ),especially for 82% of subjects with multibacillary leprosy, 10.67% of subjects with grade 2 WHO hand disability, and 9.33% of subjects with grade 2 WHO foot disability. | "We found 82% (95% CI: 75.08%-87.32%) of subjects with multibacillary leprosy, 10.67% (95% CI: 6.67%-16.62%) of subjects with grade 2 WHO hand disability, and 9.33% (95% CI: 5.64%-15.06%) of subjects with grade 2 WHO foot disability"  We have added the reviewer suggestion to our manuscript |
| 2 | There appropriate use of tables and figures to display data but some table (table 2  result showed mathematical error for variable WHO Hand Disability 150 but 148 | It has been revised according to our data, and marked in manuscript  "Grade 2 Hand Disability previously 14, has been corrected to 16" |
| 3 | Statistical analyses appropriate, and are the results interpreted correctly with missing confounding factors | We have marked the adjusted OR from the binary logistic calculation in our manuscript |
| 4 | Potential confounding factors not addressed adequately |  |
| 5 | Bivariate analyses provide a limited perspective on the complexity of real-world phenomena. To gain a more comprehensive understanding, researchers often need to employ multivariate analyses that consider multiple variables simultaneously. Bivariate analyses have certain limitations and drawbacks that should be considered when interpreting their results. Here are some common drawbacks: | correlation analysis has its pitfalls in defining a relationship between two variables, such as the results cannot be interpreted as causal or agreement between methods, the method only incorporates two variables, and the result excludes the influence of confounding variables [81]. Therefore, we have applied confounder adjustment methods such as binary logistic regression and multivariable regression analysis to provide more objective results. We acknowledge that the multivariable linear regression models could not fit for zero collinearity assumption due to analyzing variables within a variable (SALSA-work, -dexterity, and -mobility within total SALSA scale) and thus may become overfitting. However, the inclusion of these variables together is considered essential in reflecting activity limitations in the study population. Another drawback of our model is that the linear assumption was not met. However, the transformation of the result, which is also beyond the scope of this study, requires the removal of outliers and thus may create selection bias |
| 6 | Bivariate analyses focus on the relationship between two variables only, neglecting the potential influence of other variables. Real-world phenomena are often influenced by multiple factors, and the exclusion of relevant variables can oversimplify the understanding of the relationship. |  |
| 7 | Bivariate analyses may fail to account for confounding variables, which are third variables that can affect the observed relationship between the two variables being studied. Without controlling for these confounding variables, the true relationship between the variables of interest may be distorted. |  |
| 8 | Bivariate analyses, particularly correlation analyses, can show the strength and direction of a relationship but do not establish causation. Correlation does not imply causation, and there may be underlying factors or unobserved variables influencing both variables in the analysis. |  |
| 9 | well addressed but The authors should clearly discuss the limitations associated with the smaller sample size and how these limitations might introduce the roles of chance into the results. Discussing the potential impact of these limitations on the generalizability and interpretation of the findings would be valuable with explanation of analysis | The author has attached some of the limitations that may arise from this research, and described as clearly as possible |

We have answered all the feedback from the reviewers. This manuscript is not under consideration elsewhere. All authors have contributed significantly to the manuscript, have agreed to its submission to PLOS Neglected Tropical Disease in its present form, and will observe the terms of the License to Publish if the manuscript is accepted. There are no conflicts of interest. We hope that the manuscript will be put into consideration for publication in PLOS Neglected Tropical Disease. Thank you for your consideration.

Sincerely,

Dr. dr. Luh Karunia Wahyuni, Sp.KFR(K)

Paediatric Division, Dept. of Physical Medicine and Rehabilitation

Faculty of Medicine Universitas Indonesia
